# Dual Roles of CCN5/WISP2 in Cytosol and Secretome for Maintaining Muscle Homeostasis and Preventing Sarcopenia

**DOI:** 10.1002/jcsm.70282

**Published:** 2026-04-07

**Authors:** Zile Shen, Zhang Liu, Guowei Huang, Lian Cui, Wenhao Chen, Wenxi Dong, Xialin Yan, Peng Zhang, Zhen Yu

**Affiliations:** ^1^ Department of Gastrointestinal Surgery, Shanghai Tenth People's Hospital, School of Medicine Tongji University Shanghai China; ^2^ Department of Gastrointestinal Surgery, Shanghai East Hospital, School of Medicine Tongji University Shanghai China; ^3^ Department of Cardio‐Thoracic Surgery, Shanghai Tenth People's Hospital, School of Medicine Tongji University Shanghai China; ^4^ Department of Dermatology, Shanghai Skin Disease Hospital, School of Medicine Tongji University Shanghai China; ^5^ Department of Colorectal Anal Surgery The First Affiliated Hospital of Wenzhou Medical University Wenzhou China

**Keywords:** CCN5, lipid droplet‐mitochondria interaction, mitochondrial dysfunction, myosteatosis, sarcopenia

## Abstract

**Background:**

Sarcopenia is a progressive and systemic skeletal muscle disorder characterized by the decline of muscle mass and function. Despite its early‐stage, ‘possible sarcopenia’ has been emphasized for prompt intervention; there are currently no specific biomarkers for the diagnosis and treatment.

**Methods:**

RNA sequencing of human skeletal muscle across sarcopenia stages identified CCN5. Adeno‐associated virus (AAV) is intramuscularly injected into young C57BL/6J mice to knockdown CCN5 in skeletal muscle. Phenotypic alterations are assessed through behavioural testing, body composition analysis, oil red O staining and transmission electron microscopy. Mechanisms were investigated in C2C12 myotubes using lentiviral infection, Western blotting, immunofluorescence, cellular electron microscopy and seahorse assays. Finally, aged C57BL/6J mice received intramuscular AAV injections to overexpress CCN5 in skeletal muscle, evaluating its therapeutic efficacy against sarcopenia.

**Results:**

Clinical samples included 56 participants (48.2% female; mean age: 63.21 ± 8.76 years). By comparing gene expression in human skeletal muscle across three stages of sarcopenia, we identified CCN5 as a gene exhibiting decreased protein expression in possible sarcopenia stage (approximately 33% reduction, *p* = 0.0245), with this decline persisting into sarcopenia stage (*p* = 0.0093). In young mice, CCN5 knockdown induced a sarcopenia‐like phenotype, encompassing skeletal muscle dysfunction and myosteatosis (fat mass: *p* < 0.01; intramyocellular triglyceride: 66.02 ± 3.798 vs. 104.5 ± 8.542 μg/mg tissue, *p* < 0.01). CCN5 deficiency also impaired mitochondrial content and function, particularly by reducing lipid droplet‐mitochondrial (LD‐Mt) interaction (approximately 47% reduction, *p* < 0.05). In C2C12 cells, CCN5 knockdown disrupted lipid metabolism (particularly reduced lipid oxidation, CPT1A: approximately 56% reduction, *p* < 0.001), promoted lipid accumulation and compromised mitochondrial content and function. Mechanistically, secreted CCN5 enhanced LD‐Mt interaction and stimulated mitochondrial biogenesis by activating nuclear β‐catenin translocation to enhance FOXO3A‐dependent transcription, while intracellular CCN5 mitigated myosteatosis by inhibiting PPARγ signalling. In aged mice, CCN5 overexpression improved skeletal muscle function, reduced myosteatosis (fat mass: *p* < 0.001; intramyocellular triglyceride: 175.0 ± 11.18 vs. 92.18 ± 10.53 μg/mg tissue, *p* < 0.001) and restored mitochondrial function.

**Conclusions:**

CCN5 mitigates myosteatosis and counteracts sarcopenia by promoting mitochondrial biogenesis and enhancing LD‐Mt interactions through dual pathways, positioning it as a promising therapeutic target for muscle aging and sarcopenia.

## 1 Introduction

Sarcopenia is an age‐related disease characterized by progressive decline of skeletal muscle mass, decreased muscle strength and impaired physical function [1]. It markedly increases the risk of adverse outcomes in older adults, such as functional disability, frailty, fractures and even mortality [2]. In the latest sarcopenia guidelines, possible sarcopenia, as an early stage of sarcopenia, is identified as a crucial component of the sarcopenia progression. Early identification and intervention for possible sarcopenia are strongly recommended in clinical management [3, 4]. However, the mechanisms underlying sarcopenia remain elusive, arising from multiple interconnected mechanisms, including muscle atrophy, impaired regeneration, cellular senescence, inflammation, myosteatosis, mitochondrial dysfunction and hormonal changes [5]. These processes cumulatively result in modified body composition, characterized by reduced muscle mass and strength alongside heightened ectopic fat accumulation. Consequently, no specific biomarkers for the diagnosis and treatment of sarcopenia have been identified to date.

Skeletal muscle fat infiltration, also known as myosteatosis, refers to the pathological accumulation of adipose tissue within skeletal muscle intercellular spaces or the perimysium [6]. Recent studies demonstrate that this condition is not only intricately linked to progressive loss of muscle strength [7, 8] but may also exacerbate systemic metabolic disorders via mechanisms such as adipokine secretion, local inflammation and impaired insulin signalling, thereby perpetuating a detrimental cycle [9, 10]. Concurrently, Nachit M et al. noted that myosteatosis does not exhibit a definitive association with muscle mass, indicating that myosteatosis precedes atrophy in the progression of sarcopenia [11]. Our previous findings substantiate this perspective and confirm that myosteatosis constitutes a critical pathological alteration during the early stages of sarcopenia [12].

Cellular Communication Network factor 5 (CCN5), also known as WNT1 inducible signalling pathway protein‐2 (WISP2), a member of the CCN protein family, has recently emerged as a crucial regulator in metabolic diseases. It plays a critical role in the development and progression of obesity, type 2 diabetes and metabolic syndrome by modulating mesenchymal stem cell proliferation and differentiation, adipogenesis, and insulin sensitivity [13]. Recent study of Kim J. et al. has demonstrated that CCN5 knockout in mice leads to metabolic dysfunction, including ectopic fat deposition and insulin resistance [14]. Although overexpression of CCN5 was found to prevent insulin resistance [15], emerging evidence paradoxically links its endogenous expression in pancreatic β‐cells to the development of insulin resistance under obesogenic conditions [16], highlighting context‐dependent and potentially contradictory roles of CCN5 in metabolism. Furthermore, CCN5 regulates adipogenesis through multiple mechanisms, including promoting the proliferation of mesenchymal progenitor cells, modulating BMP4‐dependent differentiation and facilitating PPARγ‐mediated adipogenic commitment [15]. Importantly, as a secreted adipokine, CCN5 exerts autocrine, paracrine and endocrine effects, mediating inter‐tissue crosstalk between adipose tissue and other organs [17]. Consequently, CCN5 presents substantial promise as a critical therapeutic target for the prevention of myosteatosis and related metabolic disorders.

In the present study, leveraging on RNA sequencing of skeletal muscle from patients across three stages of sarcopenia (nonsarcopenia, possible sarcopenia and sarcopenia), we identified CCN5 as a potential protective gene against sarcopenia. Skeletal muscle‐specific deficiency of CCN5 in young mice resulted in a sarcopenia‐like phenotype, while mechanistic analyses elucidated its role in correcting lipid metabolism, diminishing myosteatosis and ameliorating mitochondrial dysfunction. Subsequent research evaluated whether skeletal muscle‐specific overexpression of CCN5 attenuated myosteatosis and mitochondrial dysfunction, hence endorsing its therapeutic potential for sarcopenia. Taken together, our present investigation has unveiled a previously unacknowledged role of CCN5 in sarcopenia and skeletal muscle aging, presenting novel opportunities for early diagnosis and treatment strategies.

## 2 Materials and Methods

### 2.1 Patients and Clinical Samples

Upon obtaining written consent, this study prospectively enrolled patients meeting the following criteria: (1) age > 18 years; (2) scheduled for single‐port video‐assisted thoracoscopic surgery with a postoperative pathological diagnosis of lung adenocarcinoma; and (3) availability of complete preoperative data including chest CT, pulmonary function and blood tests. The exclusion criteria were as follows: (1) emergency surgery, (2) pre‐existing hematologic diseases and (3) conversion to open thoracotomy. A total of 56 patients were included, and their demographics were presented in Table S1.

Diagnosis of possible sarcopenia and sarcopenia followed the Asian Working Group for Sarcopenia (AWGS) 2019 [3]. Specifically, possible sarcopenia was identified by low muscle strength and/or low physical performance, while the additional presence of low skeletal muscle mass was required for a diagnosis of sarcopenia. The following validated cut‐offs were applied [12]:

Low muscle mass: T12 skeletal muscle index (SMI) < 28.8 cm^2^/m^2^ for men, < 20.8 cm^2^/m^2^ for women, as quantified by CT.
Low handgrip strength: < 28 kg for men, < 18 kg for women.
Low physical performance: 6‐m gait speed < 1 m/s.

Prior to surgical procedure, intercostal muscles were harvested from the thoracoscopy insertion site (fourth or fifth intercostal space of the anterior axillary line), rinsed with PBS, and preserved according to the requirements of the relevant experiment. The Ethics Committee of Shanghai Tenth People's Hospital approved all procedures (SHSY‐IEC‐5.0/22 K103/P01), which were additionally registered in the Chinese Clinical Trial Registry (ChiCTR2200062303).

### 2.2 Construction of Skeletal Muscle‐Specific Adeno‐Associated Virus

The adeno‐associated virus 9 (AAV9) employed in this study was designed and produced by Obio Technology Co. Ltd. (Shanghai, China). The general steps of the experiment are as follows: shRNA or mRNA design based on the transcript of the CCN5 gene of mouse origin (NM_016873.2) was annealed into a double‐stranded oligonucleotide sequence, which was linked and cloned into the empty vector pcscAAV‐dMCK‐Cre‐tWPA (H21783) to obtain a knockdown AAV9 plasmid (pcscAAV‐dMCK‐miR30shRNA(CCN5)‐tWPA) or an overexpression AAV9 plasmid (pcscAAV‐dMCK‐CCN5‐3xFLAG‐tWPA). Subsequently, we employ GMP‐grade packaging auxiliary plasmids for virus packaging, followed by the collection and purification of the virus, and detect the virus titre by qPCR. The virus titre used in this study is approximately 2E+12 vg/mL.

The shRNA sequences are as follows:

shNC (hereinafter referred to as AAV‐NC): GAAGTCGTGAGAAGTAGAA;
shCCN5 (hereinafter referred to as AAV‐shCCN5): CACCCGAGTATCCAACCAGAA

### 2.3 Animals

Male C57BL/6J mice were purchased from Shanghai SLAC Laboratory Animal Co. Ltd. and housed in SPF‐level barriers. The mice were kept in a 12‐h light–dark cycle and had free access to standard rodent chow and water. Mice were categorized by age: young mice were 10–12 weeks old, whereas old mice were 20–22 months old.

Following dilution in PBS, the corresponding AAV9 virus was injected into the tibialis anterior (TA) and gastrocnemius (GA) muscles, with each mouse receiving 5E+10 vg/side of virus and 4–5 injection sites per muscle. One week later, these mice were sacrificed for further analysis. All mouse experiments in this study were approved by the Animal Experiment Ethics Committee of our institution (SHDSYY‐2024‐3230).

### 2.4 Cell Culture and Lentiviral Infection

C2C12 mouse myoblasts (ATCC, Cat# CRL‐1772, RRID:CVCL_0188, USA) were cultured in growth media (Dulbecco's Modified Eagle's Medium (Gibco, USA) with 10% foetal bovine serum (Gibco, USA) and 1% penicillin–streptomycin [Gibco, USA]) under conditions of 5% carbon dioxide and 37°C. To induce myogenic differentiation, cells at 70%–80% confluence were transferred to a differentiation medium (DMEM containing 2% horse serum), which was refreshed every 2 days, for a total of 7 days to promote myotube formation. For the in vitro senescence model, according to our previous study, growth medium supplemented with 40 g/L D‐gal was treated for 48 h [18].

Furthermore, Genomeditech Co. Ltd. (Shanghai, China) designed and synthesized short hairpin RNA (shRNA) lentivirus, shCtrl: TTCTCCGAACGTGTCACGT, shCCN5#1: CATGCTCTTGGCTGCAGTTAA, shCCN5#2: TGTGCCTGTCCTTGGACACCA. To construct stable knockdown cell lines, transfection was performed strictly according to the transfection kit provided by the supplier, and untransfected cells were screened out by the growth medium containing puromycin.

To block cell protein secretion, growth medium containing 5 μg/mL Brefeldin A (BFA, MCE, USA) was added for 4 h when cell density reached 70%. Additionally, to observe the effect of the gene on lipid droplets (LDs) within cells more clearly, cells were treated with growth medium containing 0.25 mM palmitate (PA) for 24 h.

### 2.5 Others

All details of the methods are shown in the Supporting Information.

## 3 Results

### 3.1 Identification of CCN5 as a Potential Protective Factor Against Sarcopenia

**FIGURE 1:**
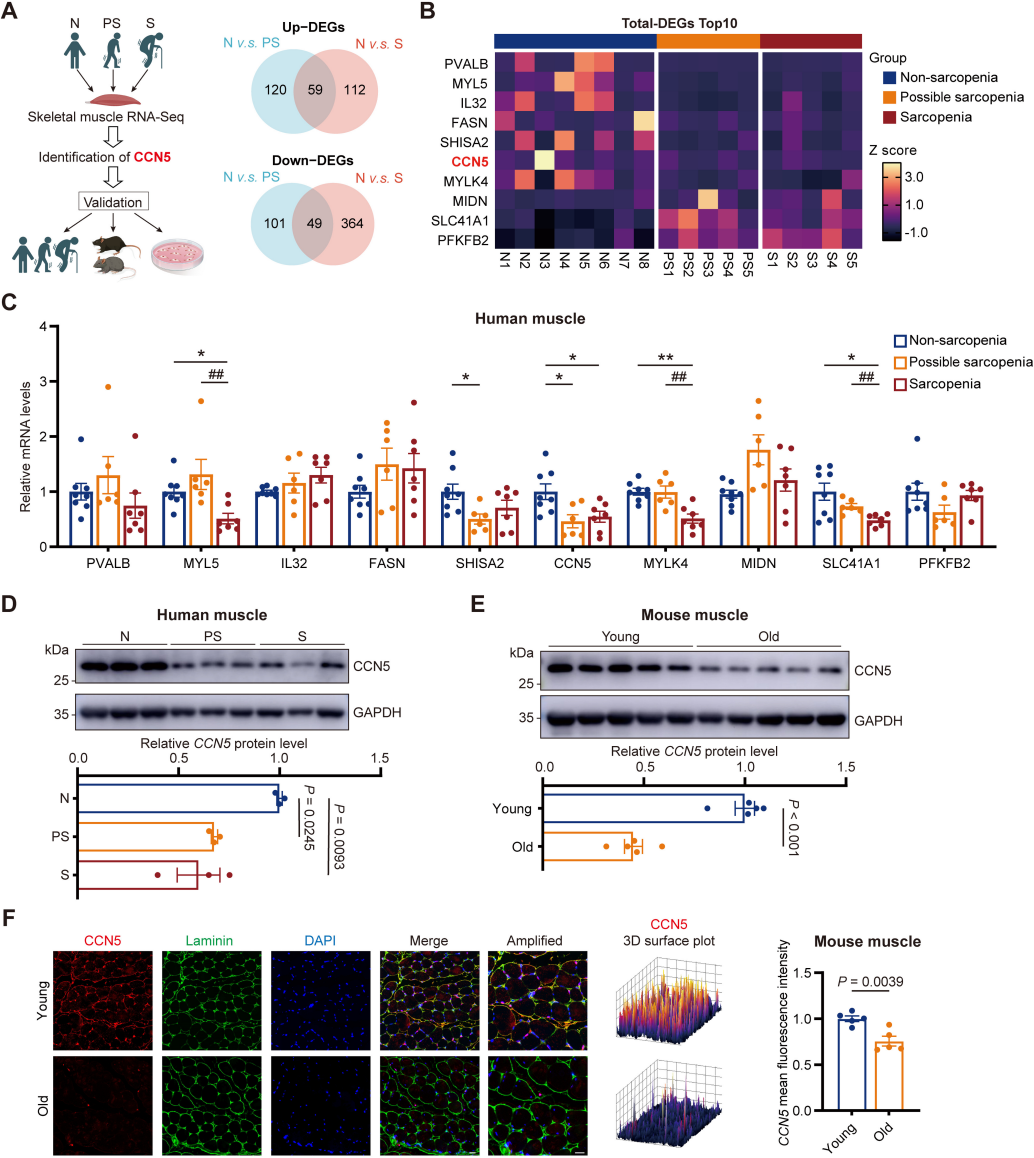
Identification of CCN5 as a potential protective factor for sarcopenia. (A) Scheme of the experimental strategy. (B) Heatmap of overlapping gene expression profile. (C) qPCR analysis of TOP 10 overlapping gene expression in N, PS, S groups (*n* = 6–8/group). (D) Relative protein expression level and quantitative data of CCN5 in human muscle (*n* = 3/group). (E) Relative protein expression level and quantitative data of CCN5 in mouse muscle (*n* = 5/group). (F) Immunofluorescence staining and enlarged images for CCN5 (red), laminin (green) and DAPI (blue) in gastrocnemius muscle sections and quantification of CCN5 levels in young and old mice (scale bars: 20 μm). The data were presented as mean ± SEM; *p < 0.05, **p < 0.01, ***p < 0.001 vs. the N group, ^#^ *p* < 0.05, ^##^ *p* < 0.01 and ^###^ *p* < 0.001 vs. the PS group. N, nonsarcopenia; PS, possible sarcopenia; S, sarcopenia.

Possible sarcopenia, a critical early stage of sarcopenia, has hitherto lacked definitive biomarkers for early diagnosis and intervention. To address this, we analysed skeletal muscle from patients at different disease stages (Figure 1A). RNA sequencing identified 329 and 584 DEGs in possible sarcopenia and sarcopenia, respectively, compared to controls (Figure S1). Focusing on genes dysregulated from the possible sarcopenia stage onward yielded 108 consistent DEGs (59 up, 49 down). From these, the top 10 candidates were selected for clinical validation (Figure 1B). Subsequent qPCR analysis of independent samples revealed that only CCN5 expression was decreased significantly from the possible sarcopenia stage onwards, remaining low in sarcopenia (Figure 1C). This distinguishes CCN5 as an early and persistent marker. Western blot confirmed the progressive decline of CCN5 protein in muscle from possible sarcopenia (*p* = 0.0245) to sarcopenia (*p* = 0.0093) (Figure 1D). A concomitant reduction in serum CCN5 was also observed in sarcopenic patients (Figure S2).

Supporting its biomarker potential, CCN5 protein and mRNA levels were also significantly reduced in aged mouse muscle (Figures 1E and S3A) and in senescent C2C12 myotubes (Figure S3B). Immunofluorescence localized CCN5 within muscle fibres and showed marked depletion in aged mice (Figure 1F). Collectively, these results establish CCN5 as a promising biomarker that declines early in sarcopenia progression.

### 3.2 Reduced CCN5 Linked Myosteatosis and Restricted LD‐Mt Interactions

**FIGURE 2:**
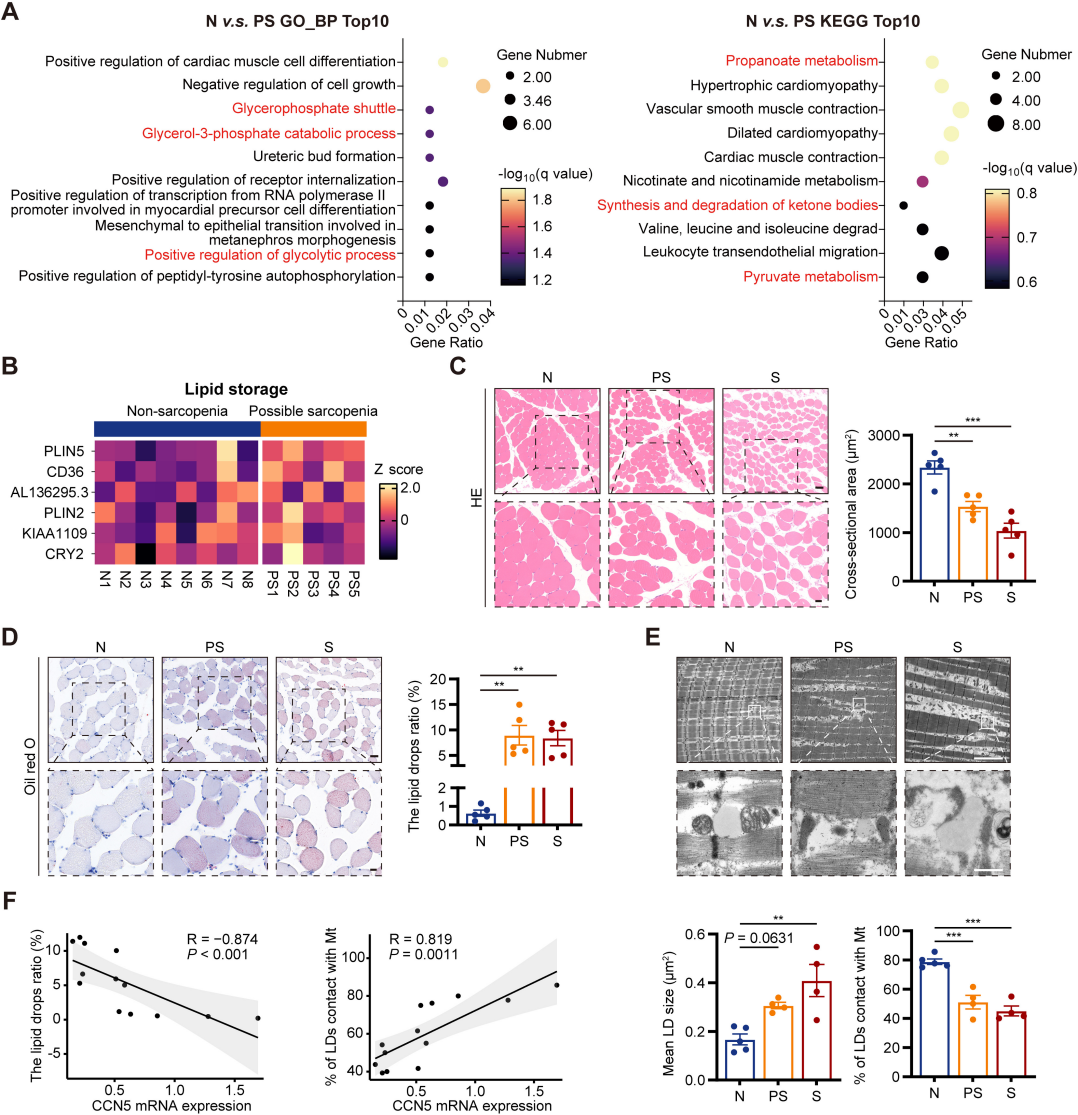
Myosteatosis, restricted LD‐mitochondria interactions and reduced CCN5 expression were observed in the skeletal muscle of possible sarcopenic patients. (A) GO and KEGG analysis of DEGs in skeletal muscle between patients with nonsarcopenia (N) and possible sarcopenia (PS). (B) Heatmap of lipid storage‐related genes in skeletal muscle from N and PS patients. (C) HE staining of human muscle and quantification of cross‐sectional area (scale bars: 50 [top] and 20 [bottom] μm). (D) Oil red O staining of human muscle and quantification of lipid droplet (LD) ratio (scale bars: 50 [top] and 20 [bottom] μm). (E) Representative TEM images of human muscle and quantification of LD (scale bars: 5 [top] and 0.5 [bottom] μm). (F) Correlation between CCN5 expression and fat infiltration index. The data were presented as mean ± SEM (*n* = 4–5/group); *p < 0.05, **p < 0.01, ***p < 0.001.

To identify early pathological changes in sarcopenia, we analysed DEGs in skeletal muscle from nonsarcopenic individuals and those with possible sarcopenia. GO and KEGG analyses revealed enrichment in skeletal muscle glucose and lipid metabolism pathways (Figure 2A), with genes involved in lipid storage notably upregulated in possible sarcopenia (Figure 2B).

At this early stage, the cross‐sectional area (CSA) of muscle fibres was already declined (Figure 2C). Myosteatosis was a crucial pathological characteristic in the progression of sarcopenia. Oil red O staining revealed a significant rise in the ratio of LDs within muscle fibres at the possible sarcopenia stage, which persists into the sarcopenia stage (Figure 2D). Electron microscopy further corroborated the changes in LDs and mitochondria inside muscle fibres (Figure 2E). Compared with nonsarcopenic individuals, patients with possible sarcopenia demonstrated a tendency for increased LD size in skeletal muscle (*p* = 0.0631), whereas sarcopenic patients displayed a significant enlargement of LDs. Analysis of mitochondrial ultrastructure revealed progressive damage. Mitochondria in the possible sarcopenia group showed irregular morphology with loss of cristae, while membranes remained largely intact. In contrast, sarcopenia was characterized by more severe swelling, cristolysis and frequent disruption of mitochondrial membranes. More importantly, the proportion of LDs in contact with mitochondria was dramatically reduced in the skeletal muscle of both possible sarcopenic and sarcopenic patients. Subsequent analysis showed that CCN5 mRNA expression exhibited an adverse correlation with the ratio of LDs in muscle fibres (R = −0.874, *p* < 0.001) and was positively correlated with the proportion of LDs in contact with mitochondria (*R* = 0.819, *p* = 0.0011; Figure 2F). In all, these data indicated that myosteatosis was an early event in sarcopenia, correlated with CCN5 expression.

### 3.3 Skeletal Muscle‐Specific CCN5 Knockdown Induced Sarcopenia‐Like Phenotypes in Young Mice

**FIGURE 3:**
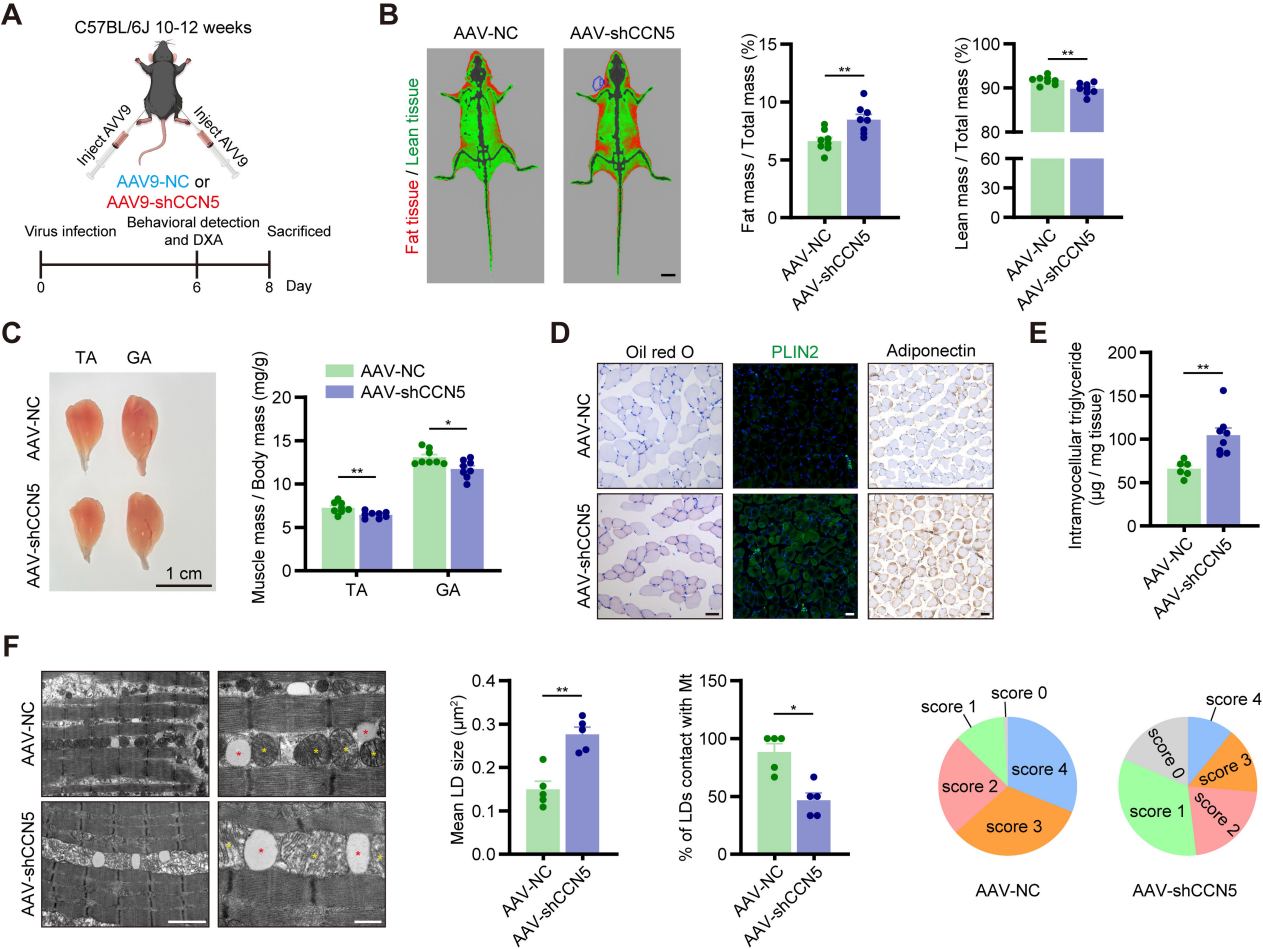
AAV9‐shCCN5‐mediated CCN5 downregulation produced sarcopenia‐like phenotype in young mice, especially myosteatosis. (A) Scheme of the experimental strategy. (B) Representative images and quantitative data of body composition measurements by dual X‐ray absorptiometry (Green, lean tissue; Red, fat tissue. Scale bars: 1 cm). (C) Representative images and muscle mass of the tibialis anterior (TA) and gastrocnemius (GA). (D) Oil red O staining, immunofluorescent staining of PLIN2 and immunohistochemical staining of adiponectin of GA muscle (scale bars: 50 μm). (E) Total triglyceride concentration in TA muscle. (F) Representative TEM images of lipid droplet (LD) and mitochondria (Mt) in GA muscle and quantification of LD, Mt. (scale bars: 2 [left] and 0.5 [right] μm). The data were presented as mean ± SEM (*n* = 5–8/group); *p < 0.05, **p < 0.01, ***p < 0.001.

To investigate the impact of CCN5 on skeletal muscle in vivo, we knocked down its expression in the TA and GA muscles of young mice utilizing an AAV9 system (Figure 3A). Successful knockdown was confirmed by reduced CCN5 mRNA and protein levels (Figure S4A). Various forced exercise indicators were used to evaluate the physical function of the two groups. AAV‐shCCN5 mice displayed significantly inferior performance, as evidenced by decreases in grip strength, hanging time, treadmill running distance and rotarod performance (Figure S4B).

Body composition analysis by dual‐energy X‐ray absorptiometry (DXA) revealed that skeletal muscle‐specific CCN5 knockdown led to augmented fat mass and diminished lean mass (Figure 3B). Consistent with this, the wet weights of TA and GA muscles were lower in AAV‐shCCN5 mice (Figure 3C). Subsequent HE and WGA staining further elucidated the basis for the difference in muscle weight, with the skeletal muscle fibres in AAV‐NC mice being approximately 15% larger than those in AAV‐shCCN5 mice (Figure S4C). Additionally, CCN5 deficiency promoted intramuscular lipid accumulation, as evidenced by increased Oil Red O staining, PLIN2 and adiponectin signal, and elevated triglyceride content in TA muscle (Figure 3D,E). Ultrastructural analysis by transmission electron microscopy (TEM) showed notably enlarged LDs and decreased proportion of LDs contacting mitochondria in CCN5 knockdown muscles (Figure 3F). Furthermore, we observed that the AAV‐shCCN5 group exhibited reduced mitochondrial quantity and quality (Figures 3F and S4D). Succinate dehydrogenase (SDH) staining (Figure S4E) and ROS fluorescence staining (Figure S4F) also verified that CCN5 deficiency intensified mitochondrial dysfunction in skeletal muscle.

To eliminate individual variability, we conducted an additional series of experiments in which AAV‐NC and AAV‐shCCN5 were injected into the contralateral TA and GA of the same mouse (Figure S5A). Consistent with the results in Figures 3 and S4, muscle injected with AAV‐shCCN5 exhibited skeletal muscle atrophy (Figure S5B,C), myosteatosis (Figure S5D–F) and mitochondrial dysfunction (Figure S6). Taken together, CCN5 deficiency contributed to myosteatosis and mitochondrial dysfunction in skeletal muscle, leading to sarcopenia‐like phenotypes.

### 3.4 CCN5 Deficiency Promoted Lipid Accumulation in C2C12 Cells by Impairing Lipid Metabolism

**FIGURE 4:**
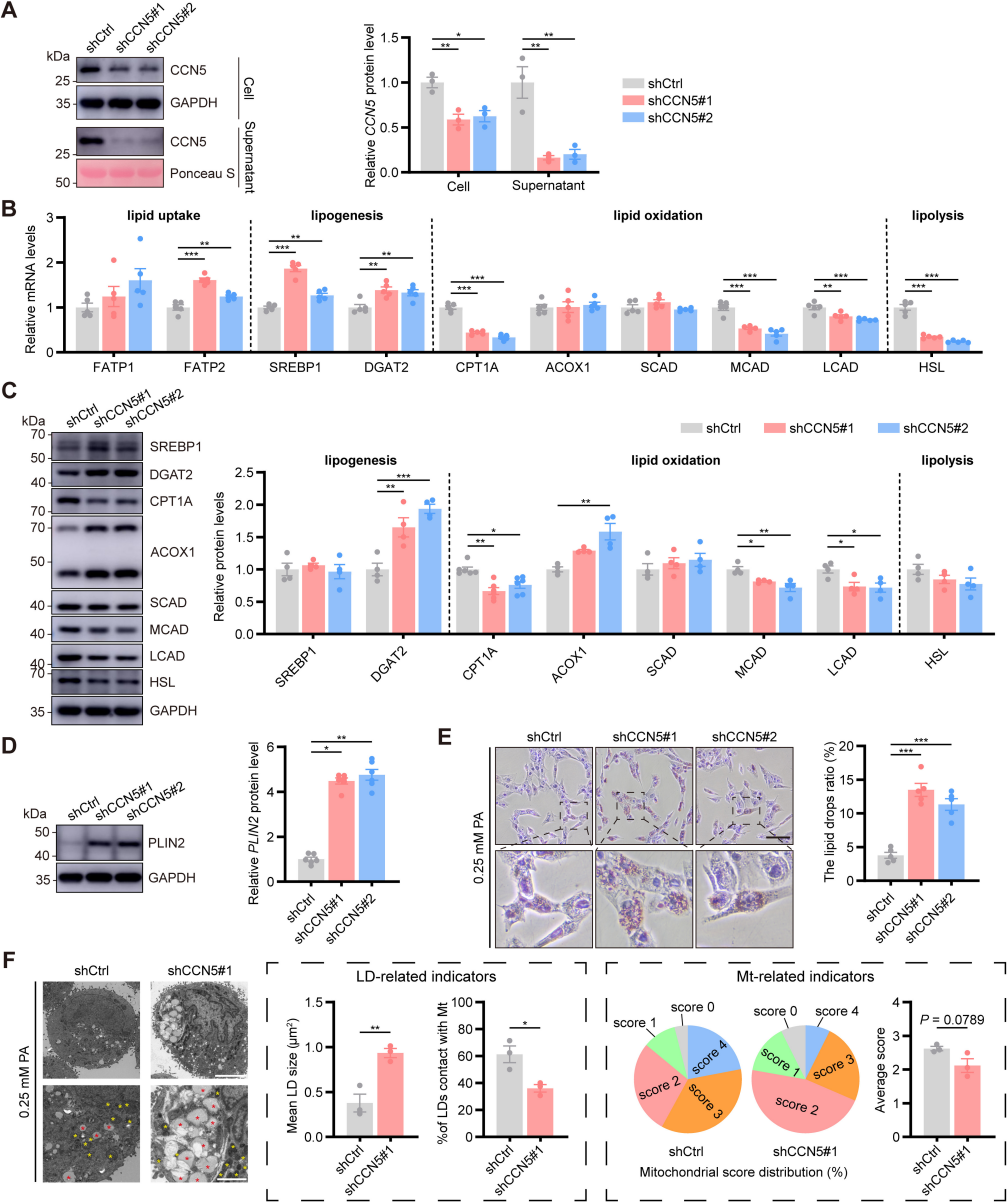
CCN5 deficiency impaired lipid metabolism and induced lipid accumulation in C2C12 cells. (A) Relative protein expression levels and quantitative data of CCN5 in intracellular and supernatants of C2C12 cells (*n* = 3/group). (B) Relative mRNA expression levels of genes related to lipid metabolism (*n* = 5/group). (C) Relative protein expression levels and quantitative data of genes related to lipid metabolism (*n* = 4–6/group). (D) Relative protein expression levels and quantitative data of PLIN2 (*n* = 6/group). (E) Oil red O staining of C2C12 cells under 0.25‐mM palmitate (PA) treatment and quantification of lipid droplet (LD) ratio (*n* = 5/group; scale bars: 100 μm). (F) Representative TEM images of LD and mitochondria (Mt) in C2C12 cells with 0.25‐mM PA treatment (left panel) and quantification of LD‐related indicator (middle panel), Mt‐related indicator (right panel) (*n* = 3/group; LD, red asterisk; Mt., yellow asterisk; scale bars: 5 [top] and 2 [bottom] μm). The data were presented as mean ± SEM; *p < 0.05, **p < 0.01, ***p < 0.001.

We established a stable CCN5‐knockdown cell line using lentiviral infection to investigate the biological role of CCN5 in lipid metabolism and accumulation in skeletal muscle. The knockdown efficiency was validated by RT‐qPCR (Figure S7A) and western blot analysis (Figures 4A and S7B). Additionally, ELISA detection of secreted CCN5 protein concentrations in the cell culture supernatant further validated the notable knockdown efficiency of the stable cell line (Figure S7C).

Analysis of lipid metabolic genes by qPCR revealed that CCN5 knockdown augmented the mRNA levels of the lipid uptake‐related gene (FATP2) and lipogenesis‐related genes (SREBP1, DGAT2) but markedly reduced the expression of lipid oxidation‐related genes (CPT1A, MCAD and LCAD) and lipolysis‐related gene (HSL) (Figure 4B). Following western blot analysis yielded consistent results, with a pronounced reduction in CPT1A expression in the knockdown group that deserves particular attention (Figure 4C).

Moreover, we noted that CCN5 deficiency increased the protein levels of PLIN2, an indicator of cellular lipid deposition (Figure 4D). To robustly visualize and quantify this phenotype, we performed staining in the presence of PA. Oil Red O and Nile red staining directly revealed a significant increase in LDs in shCCN5 cells (Figures 4E and S7D). CCN5 knockdown under PA treatment further exacerbated lipid peroxidation, as evidenced by increased BODIPY fluorescence and elevated MDA levels (Figure S8). TEM analysis showed an elevation in LDs, notably enlarged LD size and a substantial decrease in LDs contacting mitochondria within the shCCN5#1 group (Figure 4F). Further intracellular mitochondrial scoring indicated significantly reduced mitochondrial scores in shCCN5#1 cells (Figure 4F). These results collectively demonstrated that CCN5 deficiency disrupted lipid metabolism and facilitated lipid accumulation in C2C12 cells, presumably due to impaired lipid droplet–mitochondria (LD‐Mt) interactions and mitochondrial function.

### 3.5 CCN5 Expression Compromised Mitochondrial Function in C2C12 Cells, Especially Fatty Acid Oxidation

**FIGURE 5:**
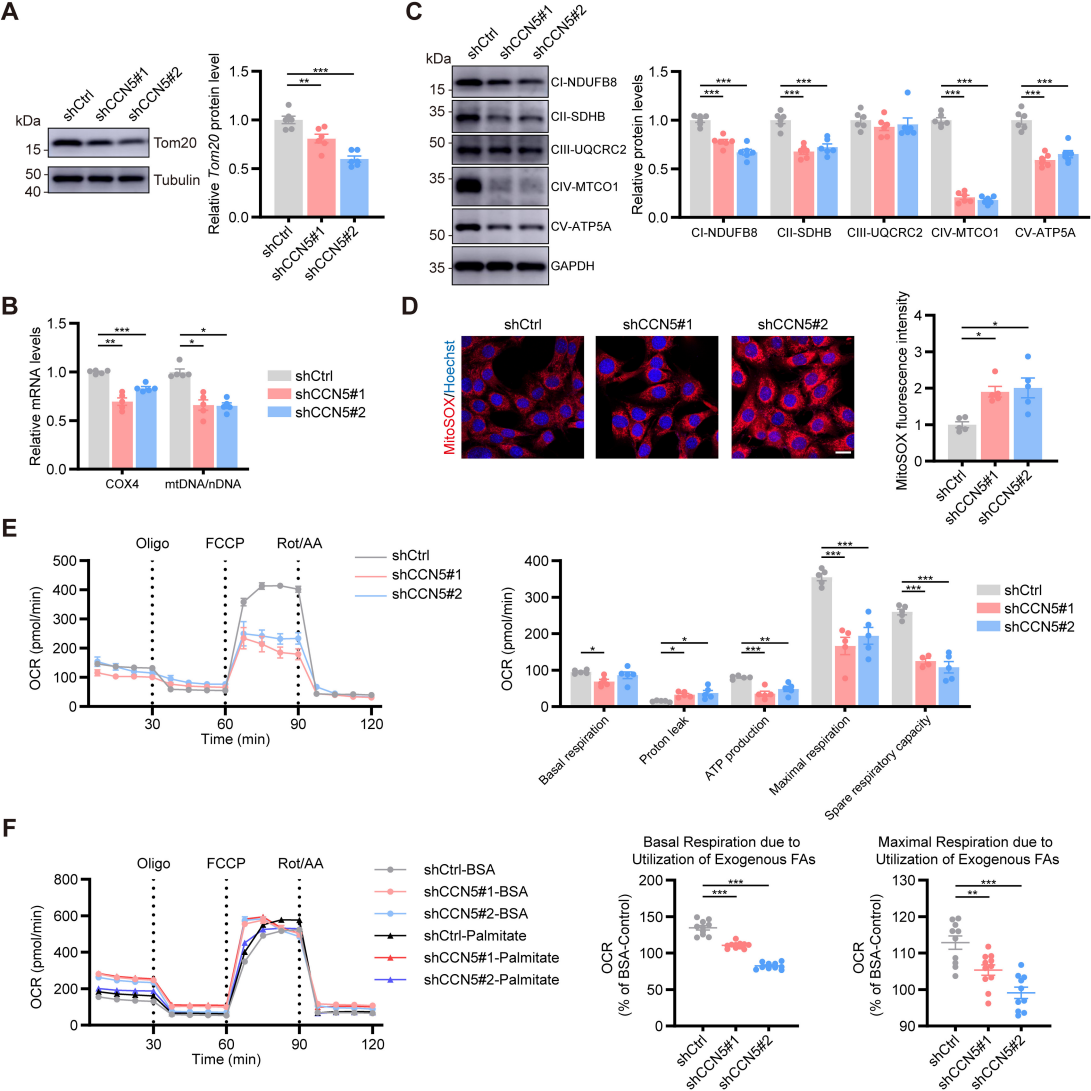
CCN5 expression affected mitochondrial function in C2C12 cells, especially fatty acid oxidation. (A) Relative protein expression level and quantitative data of Tom20 (*n* = 6/group). (B) Relative mRNA expression levels of COX4 and mitochondrial DNA copy number (mtDNA) normalized by nuclear DNA (nDNA) (*n* = 5/group). (C) Relative protein expression level and quantitative data of OXPHOS (*n* = 6/group). (D) Representative images of MitoSOX staining and quantification of fluorescence intensity (*n* = 5/group; scale bars: 20 μm). (E) Oxygen consumption rate (OCR) and statistical analysis in the indicated C2C12 cells (*n* = 5/group). (F) OCR curve of each group with or without exogenous palmitate and statistical analysis (*n* = 10–11/group). The data were presented as mean ± SEM; *p < 0.05, **p < 0.01, ***p < 0.001.

Given the mitochondrial alterations observed by TEM, we investigated whether CCN5 regulates mitochondrial function. CCN5 knockdown significantly reduced the expression of the mitochondrial protein Tom20 (Figure 5A). Indicators of mitochondrial content, including COX4 expression and the mtDNA/nDNA ratio, were also markedly decreased (Figure 5B), consistent with diminished Mitotracker fluorescence intensity (Figure S9A).

Further assessment of mitochondrial function revealed that CCN5 deficiency reduced ATP levels (Figure S9B) and decreased citrate synthase content and activity (Figure S9C,D). The protein expression of oxidative phosphorylation (OXPHOS) complexes (except complex III) was also significantly lowered (Figure 5C). MitoSOX staining indicated elevated mitochondrial ROS in knockdown cells (Figure 5D). The oxygen consumption rate (OCR) in each cell group was assessed additionally. Compared to shCtrl cells, CCN5 deficiency resulted in a modest decline in basal respiration, alongside substantial reductions in ATP production, maximal respiration and spare respiratory capacity, accompanied by increased proton leak (Figure 5E). To examine the effect of CCN5 on cellular fatty acid oxidation, we administered exogenous BSA or palmitate‐BSA as energy substrates. Interestingly, both basal and maximal respiration were significantly reduced in shCCN5#1 and shCCN5#2 cells, signifying impaired fatty acid oxidation capacity (Figure 5F). Additionally, CCN5 knockdown concurrently activated apoptotic (cleaved Caspase‐3) and senescence‐like (p21) pathways, indicating a comprehensive deterioration of cellular homeostasis (Figure S10).

### 3.6 Secreted CCN5 Boosted LD‐Mt Interactions and Mitochondrial Biogenesis via the β‐Catenin/FOXO3A Activation

**FIGURE 6:**
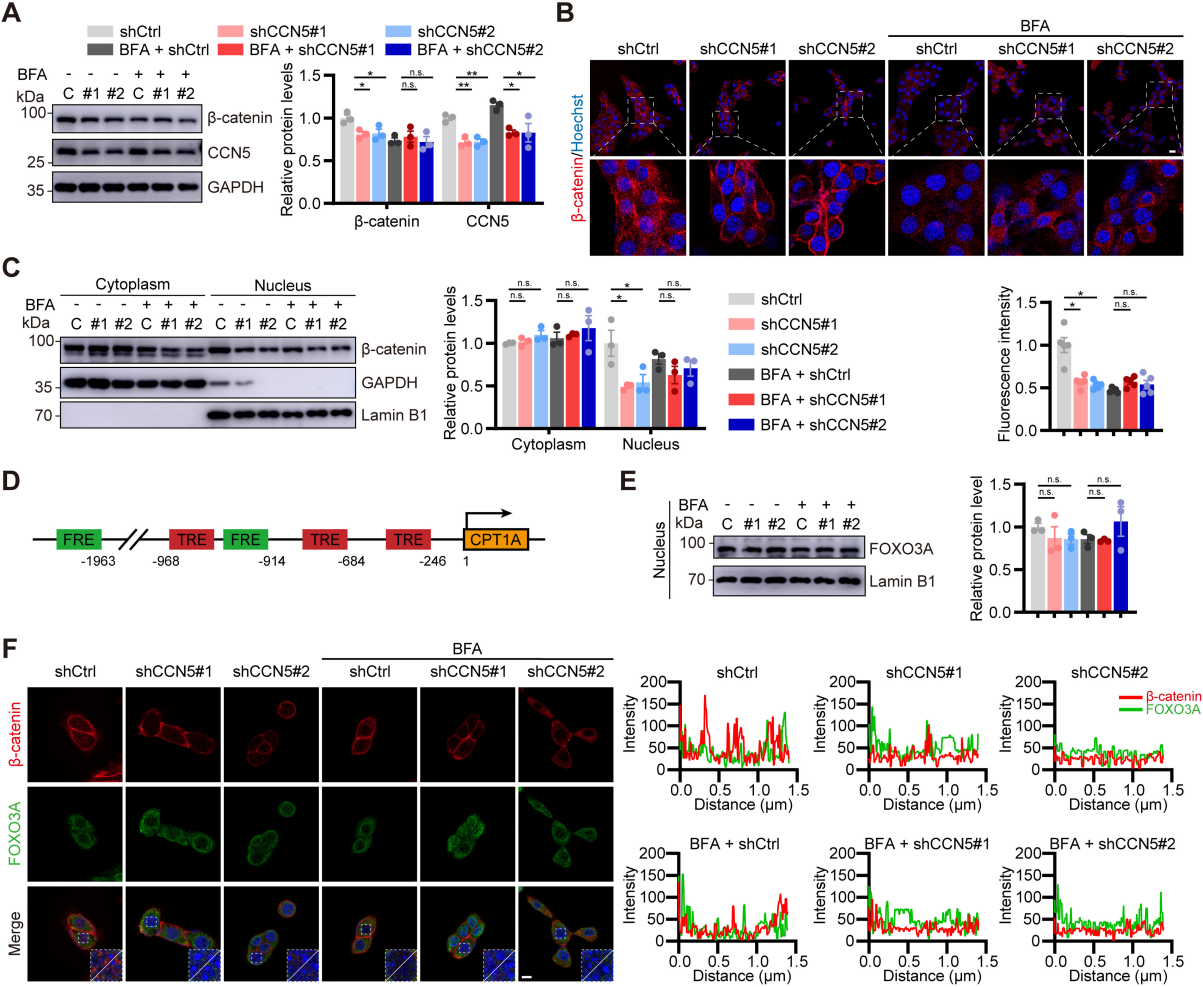
Secreted CCN5 enhanced FOXO3A‐dependent transcription by activating β‐catenin, whereas intracellular CCN5 did not. (A) Relative protein expression levels and quantitative data of β‐catenin and CCN5 (*n* = 3/group). (B) Immunofluorescent staining of β‐catenin (red) and quantification of fluorescence intensity (*n* = 5/group; scale bars: 20 μm). (C) Nucleocytoplasmic separation assay showing the changes in the intracellular localization of β‐catenin after changes in CCN5 expression (*n* = 3/group). (D) Schematic illustration of the core promoter structures of CPT1A. (E) Nucleus FOXO3A expression and quantification in each group (*n* = 3/group). (F) Co‐immunofluorescence staining of β‐catenin (red) and FOXO3A (green) (scale bars: 10 μm). The data were presented as mean ± SEM; n.s., not significant; *p < 0.05, **p < 0.01, ***p < 0.001.

CCN5 is a constituent of the Wnt/β‐catenin signalling pathway, which is acknowledged as critical for skeletal muscle regeneration. Using BFA to obstruct protein secretion, we found that although both intracellular and secreted CCN5 were diminished upon knockdown, β‐catenin protein expression only decreased in the absence of BFA. This reduction was reversed by BFA treatment (Figure 6A). Consistent results were seen from β‐catenin IF staining, showing that CCN5 knockdown reduced β‐catenin nuclear translocation (Figure 6B). Nuclear‐cytoplasmic fractionation subsequently demonstrated that secreted, but not intracellular, CCN5 promoted β‐catenin nuclear accumulation without altering cytoplasmic levels (Figure 6C).

Given CCN5's strong regulation of CPT1A (Figure 4B,C) and its role in LD‐Mt interactions, we analysed the CPT1A promoter. We identified adjacent FOXO3A recognition elements (FREs) and Tcf recognition elements (TREs), implying potential co‐activation by FOXO3A and β‐catenin/Tcf (Figure 6D). While nuclear FOXO3A protein levels were unchanged (Figure 6E), β‐catenin and FOXO3A showed nuclear co‐localization (Figure 6F), indicating that secreted CCN5 enhances CPT1A transcription by promoting β‐catenin nuclear translocation to cooperate with FOXO3A.

Subsequently, we analysed the mRNA levels of genes dependent on FOXO3A. qPCR analysis indicated a significant reduction in the expression of PGC1α, a key regulator of mitochondrial biogenesis, in shCCN5 cells relative to shCtrl cells (Figure S11A). Since CPT1A was reported to interact with PLIN2 on LDs to facilitate LD‐Mt interactions [19] (Figure S11B), AlphaFold predictions further corroborated a protein–protein interaction between CPT1A and PLIN2 (Figure S11C). This indicated that secreted CCN5 enhances CPT1A transcription by promoting β‐catenin nuclear translocation, therefore influencing lipid metabolism via LD‐Mt interactions.

### 3.7 Intracellular CCN5 Suppressed Muscle Fat Infiltration by Inhibiting PPARγ Signalling

**FIGURE 7:**
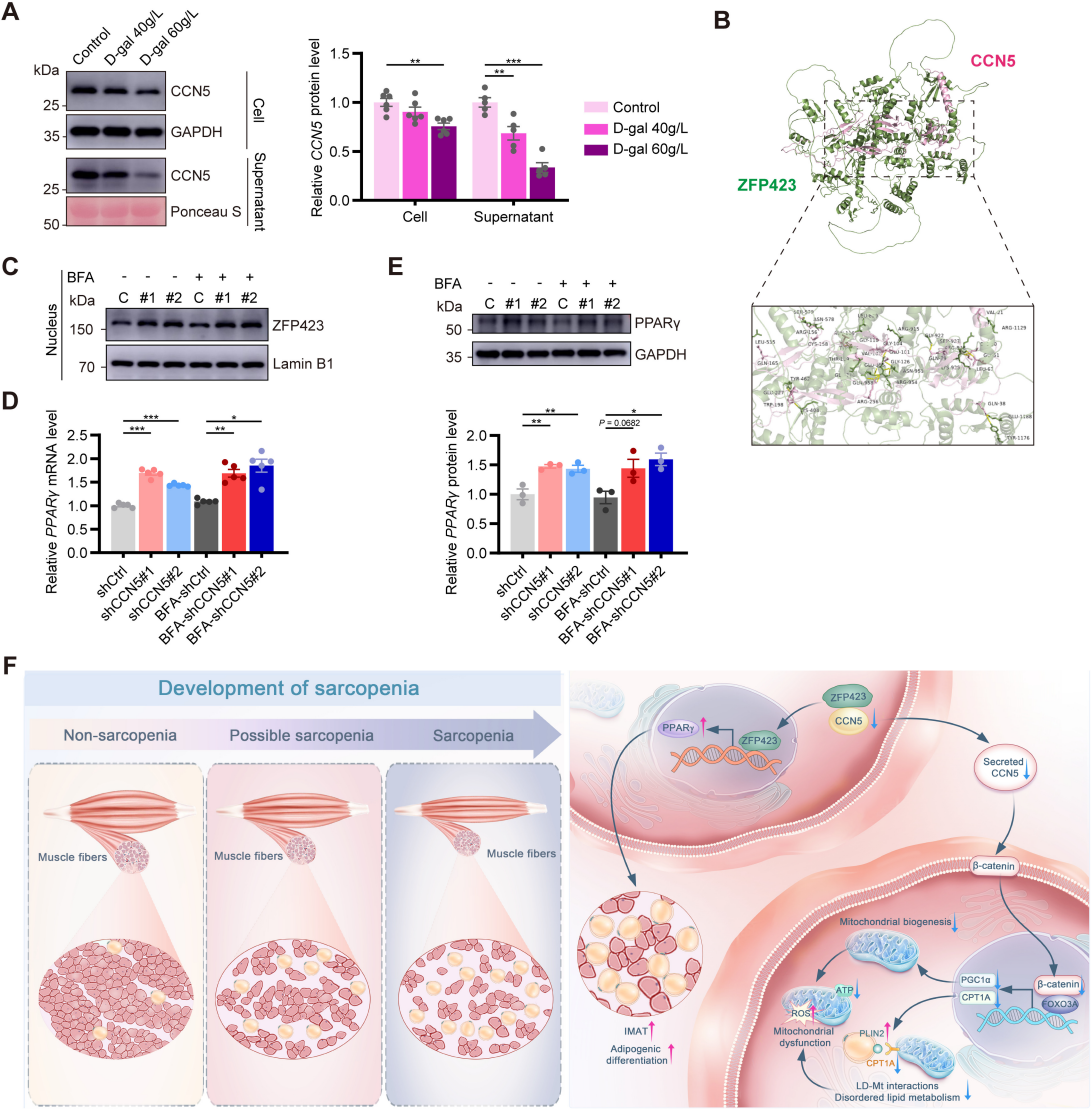
Intracellular CCN5 alleviates myosteatosis by inhibiting the PPARγ signalling pathway. (A) Relative protein expression levels and quantitative data of CCN5 in C2C12 cells following treatment with 0, 40 and 60 g/L D‐gal for 48‐h induced cell senescence (*n* = 5–6/group). (B) Model diagram of the interaction between CCN5 and ZFP423 predicted by AlphaFold. (C) Nucleus ZFP423 expression in each group. (D) Relative mRNA expression levels of PPARγ (*n* = 5/group). (E) Relative protein expression levels and quantitative data of PPARγ in each group (*n* = 3/group). (F) Schematic model summarizing the main findings of the study. The data were presented as mean ± SEM; n.s., not significant; *p < 0.05, **p < 0.01, ***p < 0.001.

Utilizing higher concentrations of D‐gal to expedite aging resulted in a significant reduction of secreted CCN5, while intracellular CCN5 protein levels similarly reduced (Figure 7A). A previous study has shown that in preadipocytes, CCN5 forms a cytoplasmic complex with ZFP423, restraining its translocation to the nucleus to activate PPARγ [20]. We first validated the interaction between CCN5 and ZFP423 through protein–protein interaction prediction (Figure 7B). Nuclear ZFP423 protein levels were subsequently quantified across different treatment groups. The results demonstrated that CCN5 knockdown significantly increased nuclear ZFP423 expression in C2C12 cells, regardless of BFA‐induced secretion inhibition (Figure 7C). As a transcriptional activator of PPARγ, augmented nuclear localization of ZFP423 is anticipated to upregulate PPARγ expression [21]. Consistent with this, qPCR analysis showed that CCN5 knockdown significantly increased PPARγ mRNA levels, regardless of secretion inhibition (Figure 7D). Comparable results were obtained by Western blot analysis (Figure 7E). These findings suggested that intracellular CCN5 obstructs ZFP423 nuclear translocation, thereby attenuating PPARγ signalling.

### 3.8 Restoration of CCN5 Ameliorated Sarcopenia‐Like Phenotypes in Aged Mice

**FIGURE 8:**
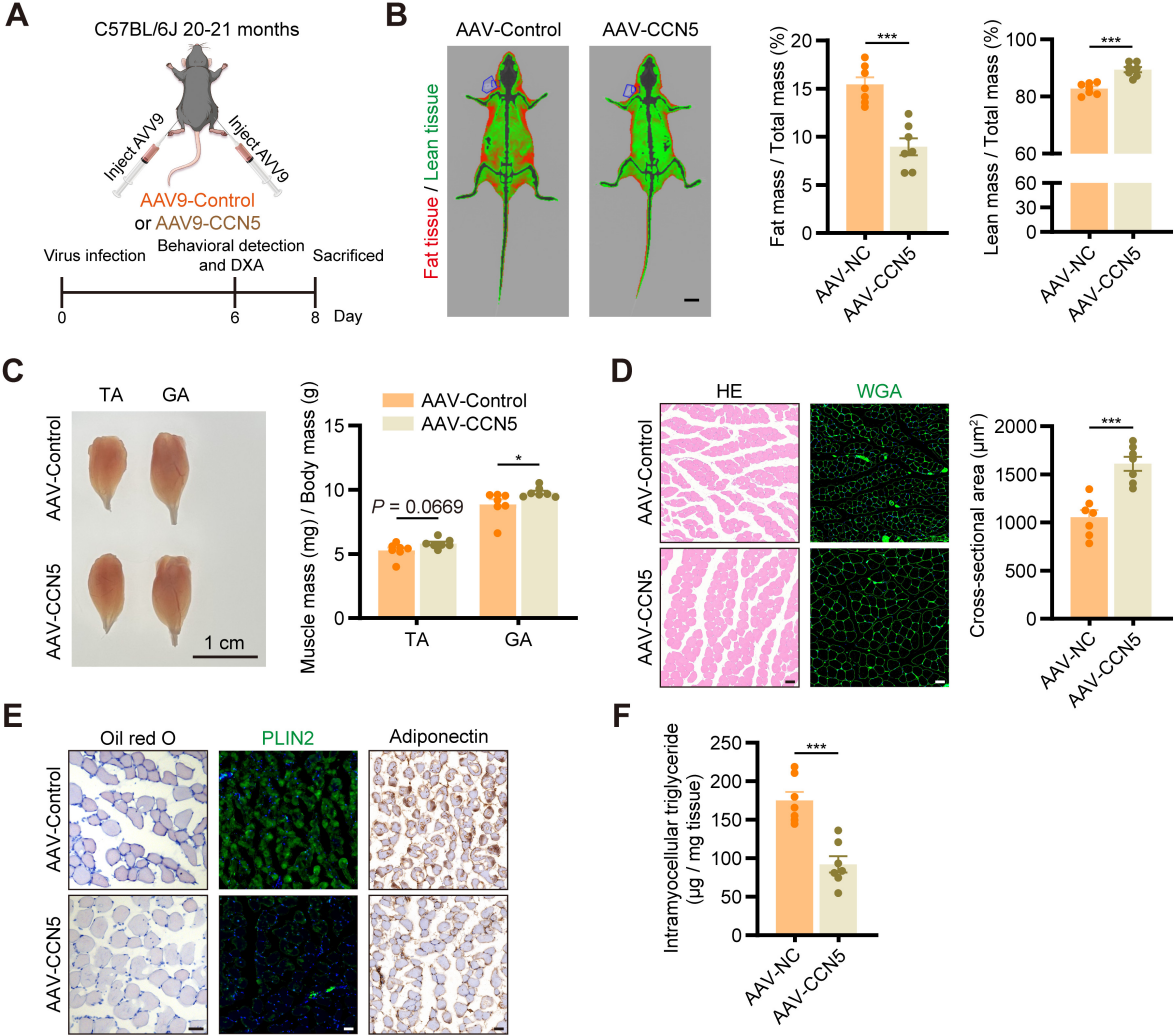
AAV9‐CCN5‐mediated CCN5 upregulation ameliorated sarcopenia‐like phenotype in aging mice, notably improving myosteatosis. (A) Scheme of the experimental strategy. (B) Representative images and quantitative data of body composition measurements by dual X‐ray absorptiometry (Green, lean tissue; Red, fat tissue. Scale bars: 1 cm). (C) Representative images and muscle mass of the tibialis anterior (TA) and gastrocnemius (GA). (D) HE and WGA staining of GA muscle and quantification of cross‐sectional area (scale bars: 50 μm). (E) Oil red O staining, immunofluorescent staining of PLIN2 and immunohistochemical staining of adiponectin of GA muscle (scale bars: 50 μm). (F) Total triglyceride concentration in TA muscle. The data were presented as mean ± SEM (*n* = 6–7/group); *p < 0.05, **p < 0.01, ***p < 0.001.

To evaluate the role of CCN5 in improving aged skeletal muscle function in vivo, 20‐month‐old mice received AAV9‐CCN5 treatment (Figure 8A), with the endpoint (Day 8 post‐injection) determined by the time‐course analysis of CCN5 mRNA expression (Figure S12A). Western blot analysis demonstrated a three‐fold elevation in CCN5 protein expression in skeletal muscle subsequent to AAV‐CCN5 injection compared to the AAV‐Control group (Figure S12B). Mice receiving AAV‐CCN5 exhibited significantly improved muscle function, as evidenced by elevated maximum grip strength, prolonged hanging time, extended treadmill test distance and higher maximum speed in rotarod (Figure S12C). Additionally, AAV‐CCN5 treatment reduced total fat mass and increased muscle mass in aged mice (Figure 8B,C). The CSA of GA was larger (Figure 8D), and myosteatosis was diminished in the AAV‐CCN5 group (Figure 8E,F). Furthermore, mitochondrial function was improved, indicated by elevated SDH activity and reduced ROS generation (Figure S12D,E). Altogether, these results elucidated that CCN5 could reverse sarcopenia‐like phenotypes in aged mice.

## 4 Discussion

Early identification, prevention and intervention play essential roles in the management of sarcopenia [3, 4]. The AWGS 2019 guidelines introduced the concept of ‘possible sarcopenia’ as an early pathological condition, characterized by low skeletal muscle strength or impaired physical performance [3]. A recent community‐based study has demonstrated that the prevalence of possible sarcopenia significantly exceeds that of sarcopenia, with rates approaching 46% [22]. More notably, a 12‐year cohort study on sarcopenia trajectories revealed that a considerable proportion of individuals with possible sarcopenia skip the overt sarcopenia stage and progress directly to mortality [23]. To our knowledge, this study represents the first investigation using human skeletal muscle tissue to identify CCN5 as a potential protective gene against sarcopenia. Our findings confirm that CCN5 expression is significantly deficient during the stage of possible sarcopenia and remains suppressed in sarcopenia, implicating its involvement in both the initiation and progression of the disease.

Growing evidence suggests that myosteatosis is an early event in sarcopenia and plays an essential part in its pathogenesis [24]. With aging, chronic inflammation or glucocorticoid exposure can redirect skeletal muscle satellite cells and fibroadipogenic progenitors from a myogenic fate toward adipogenic or fibrotic lineages [25]. Myosteatosis has been demonstrated to advance with age, irrespective of alterations in muscle mass [11], and potentially mediating subsequent atrophy [12]. Our previous large‐scale study further demonstrated that myosteatosis may be a more precise prognostic predictor than sarcopenia alone [26]. Here, we noted that diminished CCN5 expression was strongly associated with the degree of myosteatosis. The induction of CCN5 knockdown in young mice recapitulated a sarcopenia‐like phenotype characterized by considerable myosteatosis. In contrast, CCN5 overexpression in aged mice mitigated these features, highlighting the essential role of CCN5 in combating myosteatosis and its potential therapeutic value in ameliorating sarcopenia.

Lipid uptake in healthy tissue is contingent upon metabolic demand and lipid availability. Excess fatty acids are sequestered as LDs when uptake exceeds consumption [27]. In this study, sequencing of human samples revealed altered expression of lipid metabolism genes in possible sarcopenic patients compared to nonsarcopenic controls. This early metabolic dysregulation likely interacts with other established pathological features of sarcopenia, such as elevated systemic inflammation (reflected in our cohort by markers including CRP and CAR; Table S1). Consistent with Kim J et al. [14], our data demonstrated that CCN5 deficiency disrupts lipid metabolism mainly due to impaired lipid oxidation. Growing proof indicates that defective lipid oxidation is a fundamental mechanism underlying myosteatosis and sarcopenia [28]. We observed that CCN5 deficiency led to a substantial downregulation of CPT1A expression among the proteins regulating lipid oxidation. As a crucial enzyme situated on the mitochondrial outer membrane, CPT1A serves as the rate‐limiting factor in the carnitine shuttle system, facilitating the transport of long‐chain fatty acids (LCFAs). Specifically, CPT1A catalyses the transesterification of LCFAs to acylcarnitines, enabling their translocation into the mitochondrial matrix for β‐oxidation [29]. Moreover, reduced expression of MCAD and LCAD further impaired fatty acid oxidation. Collectively, these results suggest that CCN5 modulates myosteatosis by regulating the lipid oxidative metabolism of LCFAs.

Mitochondria serve as a pivotal center for energy metabolism and cellular fate regulation. Their reduced abundance, functional deficits and impaired quality control are collectively implicated in the onset and progression of sarcopenia [30]. Here, we demonstrated that CCN5 regulates mitochondrial content in skeletal muscle, likely by enhancing PGC1α expression through secreted CCN5. As a master regulator of mitochondrial biogenesis, PGC1α promotes mitochondrial DNA replication and the expression of mitochondrial‐related genes, including COX4 and NDUFB8 [31]. Consistent with this, CCN5 deficiency leads to reduced mtDNA levels and downregulation of these genes, as illustrated in Figure 5B. The concomitant activation of apoptotic (cleaved Caspase‐3) and senescence (p21) pathways upon CCN5 knockdown suggests that the profound mitochondrial dysfunction may further disrupt cellular homeostasis, contributing to a broader degenerative phenotype.

Patients with sarcopenia often exhibit dysfunctions in skeletal muscle mitochondria, characterized by impaired electron transport chain complex activity, reduced mitochondrial membrane potential and lower ATP synthesis efficiency [32]. In this study, Seahorse assay data revealed real‐time mitochondrial dysfunction following CCN5 knockdown: elevated proton leak (suggesting membrane damage), reduced maximal respiration and ATP production and critically, a decline in spare respiratory capacity (indicating insufficient functional reserve). Alongside previous observations of altered mitochondrial enzyme activities and OXPHOS complex expression, these results emphasize the essential role of CCN5 in regulating both mitochondrial content and function. While this study establishes its role in biogenesis and function, future work should explore its potential modulation of other quality control systems like dynamics and mitophagy.

Recent studies have highlighted the significance of inter‐organellar communication. Direct LD‐mitochondria contact sites enable efficient fatty acid transfer for β‐oxidation, supplying energy and reducing lipid accumulation [33]. The stability and functionality of LD‐Mt interactions rely on specific protein–protein interactions taking place at both membranes [34, 35]. A recent study identified the mitochondrial outer membrane protein CPT1A as a novel LD‐interacting protein that binds to PLIN2 on LDs, facilitating contact sites that promote LD lipolysis [19]. Consistently, we found that CCN5 knockdown downregulated CPT1A while increasing PLIN2 (presumably from lipid deposition). Importantly, impaired LD‐Mt interactions lead to the accumulation of mitochondria that are spatially separated from LDs, as evidenced by human samples, animal models and cellular experiments. This disturbance in lipid metabolism and increased fat infiltration ultimately aggravates sarcopenia.

CCN5 is a member of the CCN family, comprising six structurally conserved cysteine‐rich secreted proteins. CCN5 was originally recognized for its capacity to stimulate the proliferation of mesenchymal progenitor cells while suppressing adipogenesis and differentiation [15]. Additionally, CCN5 has been shown to activate the canonical Wnt pathway by inducing Tcf/Lef transcriptional activity and facilitating β‐catenin nuclear translocation [13]. In the present study, we further demonstrated that only the secreted CCN5 activates β‐catenin and promotes its nuclear translocation, while the cytoplasmic form does not. Previous reports indicated that β‐catenin functions as a co‐activator for transcription factors such as FOXO3A, augmenting the expression of shared target genes [36, 37]. Our findings indicate that CCN5 does not increase nuclear FOXO3A levels; the secreted CCN5 enhances β‐catenin nuclear translocation, thereby augmenting FOXO3A‐dependent transcription. This led to a marked upregulation of CPT1A and PGC1α expression. Concurrently, CCN5 is abundantly expressed in the cytoplasm, where it binds to the PPARγ transcription activator ZFP423 in mesenchymal progenitor cells [38], thereby inhibiting its nuclear translocation [20]. Comparable effects were observed in myotubes. Given that PPARγ is known to promote adipogenesis in skeletal muscle and direct myotube differentiation toward the adipocyte lineage [39], our data provide a mechanistic foundation for the myosteatosis observed in both mouse skeletal muscle and myotubes subsequent to CCN5 knockdown. In summary, our findings demonstrated that both secreted and intracellular CCN5 regulate myotubes' mitochondrial content, mitochondrial function, energy metabolism and cell fate through distinct mechanisms. Together, these regulatory actions facilitate myosteatosis, thus accelerating the progression of sarcopenia.

To date, no specific pharmacological interventions have received approval for the treatment of sarcopenia. Our current research identified CCN5 as a promising therapeutic target, and we attempted to ameliorate sarcopenia‐like phenotypes in aged mice through the exogenous administration of recombinant CCN5 protein (data not shown). However, the therapeutic outcome was inadequate, primarily due to the short half‐life of the recombinant protein, transient biological activity and ineffective targeting to skeletal muscle after systemic administration. Consequently, we employed direct skeletal muscle injection of an AAV vector overexpressing CCN5 to evaluate its functional impact in aged murine models. A promising direction for future research entails exploring strategies like structural modifications, nanocarriers or tissue‐specific promoters to attain targeted and sustained CCN5 expression for sarcopenia treatment.

Several limitations of this study should be acknowledged. The exclusive use of male mice for in vivo experiments may constrain the generalizability of our findings to females [40]. Additionally, although we used a muscle‐specific (dMCK) promoter, potential off‐target transduction within muscle cannot be fully excluded; single‐cell analyses could definitively resolve cellular origins. Furthermore, the relatively limited sample size of our clinical cohort may have reduced the statistical power for finer analyses. Future studies examining sex differences and validating CCN5 as a biomarker in prospective settings with expanded sample sizes are warranted.

In conclusion, our investigation reveals that the progression of sarcopenia is driven by diminished CCN5 levels (Figure 7F). Secreted CCN5 enhances LD‐Mt interactions and stimulates mitochondrial biogenesis by activating β‐catenin, thereby promoting FOXO3A‐dependent transcription. Intracellular CCN5, conversely, attenuates myosteatosis by inhibiting PPARγ signalling. Moreover, CCN5 overexpression reduces myosteatosis in aged murine skeletal muscle and ameliorates age‐related muscle dysfunction. These results establish a conceptual framework for developing therapeutic strategies aimed at muscle aging and sarcopenia.

## Conflicts of Interest

The authors declare no conflicts of interest.

## Supporting information


**Figure S1:** RNA sequencing of patients with nonsarcopenia (N), possible sarcopenia (PS) and sarcopenia (S). (A) Volcano plot of differentially expressed genes (DEGs) in skeletal muscle from non‐sarcopenic and possible sarcopenic patients. (B) Volcano plot of DEGs in skeletal muscle from nonsarcopenic and sarcopenic patients. (C) Heatmap of DEGs clustering expression from nonsarcopenic and possible sarcopenic patients. (D) Heatmap of DEGs clustering expression from nonsarcopenic and sarcopenic patients.
**Figure S2:** Serum levels of CCN5 from patients in N, PS, S groups (*n* = 6/group). The data were presented as mean ± SEM; *p < 0.05. N, nonsarcopenia; PS, possible sarcopenia; S, sarcopenia.
**Figure S3:** Validation of CCN5 mRNA expression levels in aged mice and aged myotubes. (A) qPCR analysis of CCN5 gene expression in muscles from indicated mice (*n* = 8/group). (B) qPCR analysis of CCN5 gene expression in young and senescent C2C12 cells (*n* = 3/group). The data were presented as mean ± SEM.
**Figure S4:** AAV9‐shCCN5‐mediated CCN5 downregulation impaired skeletal muscle function in young mice and decreased mitochondrial content and function. (A) Relative mRNA and protein expression levels of CCN5 in tibialis anterior (TA) muscle. (B) Maximal grip strength, four‐paw hanging time, total distance for the treadmill test, maximum speed for the rotarod test of the mice in various groups. (C) HE and WGA staining of gastrocnemius (GA) and quantification of cross‐sectional area (scale bars: 50 μm). (D) The number and overall average score of mitochondria (Mt) and from TEM images. (E) SDH staining of GA sections (scale bars: 100 μm). (F) Representative images of dihydroethidium (DHE) staining of GA muscle and quantitative data of reactive oxygen species (ROS) in various groups (scale bars: 50 μm). The data were presented as mean ± SEM (*n* = 5–8/group); *p < 0.05, **p < 0.01, ***p < 0.001.
**Figure S5:** CCN5 reduction produced sarcopenia‐like phenotype in young mice, especially myosteatosis. (A) Scheme of the experimental strategy. (B) Representative images and muscle mass of the tibialis anterior (TA) and gastrocnemius (GA). (C) HE and WGA staining of GA muscle and quantification of cross‐sectional area (Scale bars: 50 μm). (D) Oil red O staining, immunofluorescent staining of PLIN2 and immunohistochemical staining of adiponectin of GA muscle (Scale bars: 50 μm). (E) Total triglyceride concentration in TA muscle. (F) Representative TEM images of lipid droplet (LD) and mitochondria (Mt) in GA muscle and quantification of LD, Mt. (Scale bars: 2 (left) and 0.5 (right) μm). The data were presented as mean ± SEM (*n* = 3/group); n.s., not significant; *p < 0.05, **p < 0.01, ***p < 0.001.
**Figure S6:** CCN5 reduction contributed to impaired mitochondrial content and function in young mice. (A) The number and overall average score of mitochondria (Mt) and from TEM images. (B) SDH staining of GA sections (Scale bars: 100 μm). (C) Representative images of dihydroethidium (DHE) staining of GA muscle and quantitative data of reactive oxygen species (ROS) in various groups (Scale bars: 50 μm). The data were presented as mean ± SEM (*n* = 3/group); *p < 0.05, **p < 0.01, ***p < 0.001.
**Figure S7:** CCN5 deficiency induced lipid accumulation in C2C12 cells. (A) qPCR analysis of CCN5 gene expression in the indicated C2C12 cells (*n* = 5/group). (B) Relative protein expression levels and quantitative data of CCN5 in C2C12 cells with protein secretion blocked by 5 μg/mL Brefeldin A for 4 h (*n* = 3/group). (C) Secreted CCN5 expression level was determined by enzyme‐linked immunosorbent assay (*n* = 5/group). (D) Nile Red staining and quantification of fluorescence intensity in C2C12 cells under 0.25 mM palmitate (PA) treatment (n = 5/group; Scale bars: 200 μm). The data were presented as mean ± SEM; *p < 0.05, **p < 0.01, ***p < 0.001.
**Figure S8:** Increased lipid peroxidation in CCN5‐deficient myotubes under PA treatment. (A) BODIPY staining and quantification of fluorescence intensity in C2C12 cells under 0.25 mM palmitate (PA) treatment (Scale bars: 100 μm). (B) Quantification of lipid peroxidation by MDA assay. The data were presented as mean ± SEM (*n* = 5/group); *p < 0.05, **p < 0.01, ***p < 0.001.
**Figure S9:** CCN5 expression impacted mitochondrial content and function in C2C12 cells. (A) Representative images of Mitotracker staining and quantification of fluorescence intensity (*n* = 5/group; scale bars: 20 μm). (B) ATP content normalized by total protein content (*n* = 6/group). (C) Relative mRNA expression levels of citrate synthase (CS) (*n* = 5/group). (D) CS activity (by colorimetric method) of different groups (*n* = 5/group). The data were presented as mean ± SEM; *p < 0.05, **p < 0.01, ***p < 0.001.
**Figure S10:** Analysis of apoptosis and senescence markers following CCN5 knockdown. (A) Relative protein expression levels and quantitative data of cleaved Caspase‐3. (B) Relative protein expression levels and quantitative data of p21 and p53. The data were presented as mean ± SEM (*n* = 4/group); *p < 0.05, **p < 0.01, ***p < 0.001.
**Figure S11:** Secreted CCN5 enhanced FOXO3A‐dependent transcription. (A) Relative mRNA expression levels of genes depended on FOXO3A transcription (*n* = 5/group). (B) Model of CPT1A‐PLIN2 conjugation promoting lipid droplet‐mitochondrial interactions. (C) Model diagram of the interaction between CPT1A and PLIN2 predicted by AlphaFold. The data were presented as mean ± SEM; *p < 0.05, **p < 0.01, ***p < 0.001.
**Figure S12:** AAV9‐CCN5‐mediated CCN5 upregulation improved skeletal muscle function, enhanced mitochondrial content and function in aged mice. (A) Time‐course analysis of CCN5 mRNA expression following intramuscular AAV injection (*n* = 3/group; statistical comparison vs. Day 0). (B) Relative protein expression levels and quantitative data of CCN5 in tibialis anterior (TA) muscle. (C) Maximal grip strength, four‐paw hanging time, total distance for the treadmill test, maximum speed for the rotarod test of the mice in various groups. (D) SDH staining of GA sections (Scale bars: 100 μm). (E) Representative images of dihydroethidium (DHE) staining of GA muscle and quantitative data of reactive oxygen species (ROS) in various groups (Scale bars: 50 μm). The data were presented as mean ± SEM (*n* = 6–7/group); *p < 0.05, **p < 0.01, ***p < 0.001.
**Table S1:** Clinical data of study patients by group. SD, standard deviation; BMI, body mass index; T12 SMI, skeletal muscle index at 12th thoracic vertebra; IQR, interquartile range; SPPB, Short Physical Performance Battery; TNM, tumour‐nodule‐metastasis; CAR, C‐reactive protein‐to‐albumin ratio.
**Table S2:** The primary antibodies used for WB and IHC/IF assays.
**Table S3:** Primer sequences for RT‐qPCR analysis.
