# Supplementary material for: Dual Roles of CCN5/WISP2 in Cytosol and Secretome for Maintaining Muscle Homeostasis and Preventing Sarcopenia
Source: J Cachexia Sarcopenia Muscle. 2026 Apr 7;17(2):e70282. doi: 10.1002/jcsm.70282 (PMC13055464; doi:10.1002/jcsm.70282)
Supplement: Supplementary file 1 — Figure S1: RNA sequencing of patients with nonsarcopenia (N), possible sarcopenia (PS) and sarcopenia (S). (A) Volcano plot of differentially expressed genes (DEGs) in skeletal muscle from non‐sarcopenic and possible sarcopenic patients. (B) Volcano plot of DEGs in skeletal muscle from nonsarcopenic and sarcopenic patients. (C) Heatmap of DEGs clustering expression from nonsarcopenic and possible sarcopenic patients. (D) Heatmap of DEGs clustering expression from nonsarcopenic and sarcopenic patients. Figure S2: Serum levels of CCN5 from patients in N, PS, S groups (n = 6/group). The data were presented as mean ± SEM; *p < 0.05. N, nonsarcopenia; PS, possible sarcopenia; S, sarcopenia. Figure S3: Validation of CCN5 mRNA expression levels in aged mice and aged myotubes. (A) qPCR analysis of CCN5 gene expression in muscles from indicated mice (n = 8/group). (B) qPCR analysis of CCN5 gene expression in young and senescent C2C12 cells (n = 3/group). The data were presented as mean ± SEM. Figure S4: AAV9‐shCCN5‐mediated CCN5 downregulation impaired skeletal muscle function in young mice and decreased mitochondrial content and function. (A) Relative mRNA and protein expression levels of CCN5 in tibialis anterior (TA) muscle. (B) Maximal grip strength, four‐paw hanging time, total distance for the treadmill test, maximum speed for the rotarod test of the mice in various groups. (C) HE and WGA staining of gastrocnemius (GA) and quantification of cross‐sectional area (scale bars: 50 μm). (D) The number and overall average score of mitochondria (Mt) and from TEM images. (E) SDH staining of GA sections (scale bars: 100 μm). (F) Representative images of dihydroethidium (DHE) staining of GA muscle and quantitative data of reactive oxygen species (ROS) in various groups (scale bars: 50 μm). The data were presented as mean ± SEM (n = 5–8/group); *p < 0.05, **p < 0.01, ***p < 0.001. Figure S5: CCN5 reduction produced sarcopenia‐like phenotype in young mice, especially myosteatosis. [file JCSM-17-e70282-s001.docx]

**Supplementary Material**

**Dual Roles of CCN5/WISP2 in Cytosol and Secretome for Maintaining Muscle Homeostasis and Preventing Sarcopenia**

*Zile Shen*^#^*, Zhang Liu*^#^*, Guowei Huang, Lian Cui, Wenhao Chen, Wenxi Dong, Xialin Yan,*

*Peng Zhang^*^, Zhen Yu^*^*

**This file includes:**

Materials and Methods

Supplemental Figures S1-S12

Supplemental Tables S1-S3

**Materials and Methods**

**1.1** **RNA sequencing and bioinformatic analysis**

Total RNA was extracted from skeletal muscle of patients with non-sarcopenia, possible sarcopenia, and sarcopenia using TRIzol reagent. Subsequently, Biomarker Technologies Co., LTD. (Beijing, China) performed RNA quality control and library construction, subsequently executing sequencing on the Illumina NovaSeq6000 platform. Bioinformatic analysis was performed using BMKCloud (www.biocloud.net). Differentially expressed genes (DEGs, fold change ≥ 1.5 and *P* < 0.05) were analyzed utilizing the DESeq2 package in R. For subsequent focused analysis and visualization, the top 10 DEGs were selected based on a combined ranking of statistical significance (P value) and magnitude of change (absolute log2 fold change). The definitive identification of candidate protective genes against sarcopenia was ultimately based on validation of these candidates in independent clinical samples. Heatmaps were generated using the pheatmap package in R based on the Z scores of the DEGs. Concurrently, Gene Ontology (GO) and Kyoto Encyclopedia of Genes and Genomes (KEGG) analyses were performed using clusterProfiler to annotate the DEGs and to classify them functionally.

**1.2** **Enzyme-linked immunosorbent assay (ELISA) of the patient's serum**

Following the manufacturer's instructions, the ELISA method was utilized to quantify the serum levels of CCN5, employing the Human WISP2/CCN5 ELISA kit (YOBIBIO, Shanghai). The lower limits of detection for CCN5 was 31 ng/L.

**1.3 Detection of skeletal muscle function**

Four forced exercise methods were used to evaluate skeletal muscle function in mice: grip strength, hanging test, treadmill test, and rotarod test. The procedures have been described in previous study [1]. Prior to intramuscular injection of AAV9, the exercise capacity of mice was screened by the above four approaches, and mice with total scores between 15% and 85% were selected for subsequent experiments. Six days after injection, the skeletal muscle function of mice was re-assessed.

**1.4 Body composition analysis**

Dual-energy X-ray absorptiometry (DXA; iNSiGHT VET DXA, OsteoSys, Korea) was utilized to assess body composition. Anesthetized mice were positioned prone on the scanner bed, limbs and tail extended. Within the composition images, fat tissue is displayed in red and lean tissue in green.

**1.5 Muscle triglyceride analysis**

The triglyceride content in skeletal muscle tissue was measured by a commercial kit (Beyotime, China). In brief, isopropanol was added to grind the skeletal muscle tissue thoroughly for complete lysis. Then, after centrifugation at 12000 g for 5 min at 4°C, the supernatant was collected. A standard curve was established by the kit's standard samples to determine the triglyceride content in each sample. Finally, the triglyceride content in each sample was further standardized based on skeletal muscle mass.

**1.6 Succinate dehydrogenase (SDH) staining**

Following euthanasia, GA was embedded in OCT solution (TissueTek, Japan), snap-frozen at -20°C, and sectioned at 8 µm for further staining. Sections were incubated in SDH reactive reagent (Solarbio, China) at 37°C for 30 min, washed with PBS, and examined under a microscope (Nikon, Japan).

**1.7 DHE staining**

Frozen sections (8 μm thick) were incubated in the dark with DHE working solution (Merck, Germany) for 45 min. Subsequently, they were observed using a fluorescence microscope (Nikon, Japan). Image analysis for mean fluorescence intensity was performed by ImageJ software.

**1.8 Western blot analysis**

Skeletal muscle tissues or cells were lysed in RIPA buffer containing protease and phosphatase inhibitors to extract total protein. Upon protein quantification with a BCA assay kit (Beyotime, China), equal amounts of protein were separated by SDS-PAGE and transferred to PVDF membranes (Millipore, USA). Membranes were blocked with 5% non-fat milk for 1 h at room temperature, then incubated with primary antibodies (Table S2) overnight at 4°C, followed by secondary antibodies for 1 h at room temperature. Rinse the membrane three times with TBS/Tween buffer (Epizyme, China) to remove any unbound antibodies. Finally, visualize the protein bands using the ECL protein blotting detection system.

**1.9 RNA extraction and quantitative real-time polymerase chain reaction (RT-qPCR)**

Total RNA was extracted from skeletal muscle tissues or cultured cells using TRIzol reagent (Invitrogen, USA), followed by reverse transcription into cDNA with TOROIVD qRT Master Mix 2.0 (TOROIVD, China). RT-qPCR was then performed utilizing Hieff qPCR SYBR Green Master Mix (Yeasen, China). The primer sequences were listed in Table S3.

**1.10 Immunofluorescence (IF) and immunohistochemical (IHC) staining**

For tissue sections, in collaboration with Servicebio (Wuhan, China), paraffin-embedded skeletal muscle tissue from patients and mice was sectioned for subsequent IF and IHC staining. For cell experiments, cells were cultured on confocal dishes. After washing with PBS, the cells were fixed for 20 min, followed by permeabilization with 0.5% Triton X-100 and blocking with 5% goat serum for 1.5 h. The cells were then incubated overnight at 4°C with specific antibodies. Subsequently, the cells were incubated at room temperature for 1 h with the corresponding secondary antibody, followed by Hoechst staining (Beyotime, China) of nuclei. The antibodies used in the staining process were presented in Table S2.

**1.11 Oil red O staining**

Dissolve oil red O (Sigma, USA) powder in isopropanol (0.5 g in 100 ml) to prepare a stock solution. Mix the stock solution with H_2_O (3:2) to obtain the working solution and filter. Frozen sections of skeletal muscle tissue or cultured cells were fixed, then soaked in 60% isopropanol for 30 s, followed by immersion in the working solution for 20 min. After washing with 60% isopropanol and H_2_O, the cell nuclei are stained with hematoxylin (Beyotime, China).

**1.12 Transmission electron microscopy (TEM)**

For skeletal muscle, the tissue was cut into 1 mm × 1 mm × 2 mm strips along the fiber direction and fixed in electron microscopy fixative (Servicebio, China) at 4 °C for 12 h. While cultured cells were scraped with PBS, then fixed in the same fixative at room temperature for 30 min followed by 4 °C for 8 h. Subsequently, these samples were then post-fixed in 1% osmic acid for 2 h. After ethanol dehydration, they were infiltrated with an acetone–embedding medium mixture and embedded. Ultrathin sections (80 nm) were prepared and double-stained with uranyl acetate and lead citrate, then examined using a Hitachi TEM system (Hitachi, Japan) at 80 kV. To quantify the proportion of lipid droplets (LDs) in contact with mitochondria, five random fields per sample were analyzed at high magnification. Two independent, trained observers used ImageJ software (version 1.48, USA) to manually identify all LDs and count those physically juxtaposed to mitochondria (defined as having no visible gap between organelles). The LD-mitochondria contact proportion for each sample was calculated as the average of the two counts. A five-point scoring system, based on the content and integrity of the cristae, was utilized to assess mitochondrial morphology [2]. The specific criteria were defined as follows:

Score 4: >80% cristae content; cristae are clearly defined, densely packed, and intact.

Score 3: 60-80% cristae content; cristae are present but show mild irregularities.

Score 2: 30-60% cristae content; cristae are significantly disorganized, with discontinuous membranes.

Score 1: 10-30% cristae content; cristae are severely fragmented or swollen, with pronounced membrane distortion.

Score 0: <10% cristae content; cristae are virtually absent with severe membrane disruption.

**1.13 Protein extraction from cell culture supernatant**

When cells reached 80% confluency, the medium was switched to Opti-MEM (Gibco, USA) and cultured for another 24 h. Then, the supernatant was collected and centrifuged at 3000 rpm and 4 °C for 5 min. Methanol and chloroform were added in a 4:4:1 ratio, followed by thorough mixing and centrifugation at 13000 rpm and 4 °C for 5 min. Finally, the middle protein layer was retained, mixed with methanol, and centrifuged again at 13000 rpm and 4 °C for 10 min. The resulting protein pellet was dissolved in protein loading buffer, heated in a metal bath, and used for subsequent experiments.

**1.14 ELISA of cell culture supernatant**

CCN5 content in the cell culture supernatant was measured utilizing a commercial ELISA kit (Boyun, China). Briefly, after cells were switched to Opti-MEM and cultured for 24 h, the supernatant was collected, centrifuged at 2500 rpm and 4 °C for 5 min, and then re-collected. A standard curve was generated with the kit's standards, and the CCN5 concentration in each sample was determined by ELISAcalc software.

**1.15 Nile red staining**

According to the Nile Red reagent kit protocol (Beyotime, China), the lipid droplet staining solution was prepared by combining 1 µl Nile Red (1000X), 1 µl Hoechst (1000X), and 998 µl Assay Buffer. After fixation, the staining solution was added and incubated at room temperature in the dark for 15 min. The samples were then washed with PBS and visualized using an inverted fluorescence microscope (Nikon, Japan).

**1.16 Analysis of lipid peroxidation**

Lipid peroxidation was assessed using two complementary assays. First, the level of cellular lipid peroxidation was evaluated using the fluorescent probe BODIPY 581/591 C11 (Beyotime, China), according to the manufacturer's instructions. This ratiometric probe exhibits a shift in fluorescence emission from red to green upon oxidation, allowing for qualitative visualization. Second, the concentration of malondialdehyde (MDA), a terminal product of lipid peroxidation, was quantified using a commercial Lipid Peroxidation (MDA) Assay Kit (Beyotime, China) via the thiobarbituric acid (TBA) method, following the standard protocol.

**1.17 ATP content detection**

ATP content was quantified using an assay kit (Beyotime, China). Briefly, cells were fully lysed with lysis buffer and centrifuged at 12000 g for 5 min at 4 °C to collect the supernatant. A standard curve was generated by the provided standards to calculate ATP levels. Finally, protein concentration was determined with a BCA protein assay kit (Beyotime, China) to normalize the ATP content.

**1.18 Cell mitochondrial function assessment**

All experimental procedures were performed in strict compliance with the manufacturer's protocols. Mitochondria with intact biological activity were specifically labeled using MitoTracker Red CMXRos (Beyotime, China). Mitochondrial reactive oxygen species (ROS) levels were measured with the MitoSOX Red mitochondrial superoxide indicator (Yeasen, China). Mitochondrial enzyme activity was assessed via citrate synthase (CS) activity using a CS activity assay kit (Abbkine, China).

Additionally, the mitochondrial oxygen consumption rate (OCR) and fatty acid oxidation capacity of C2C12 myotubes were measured using the XFe96 extracellular flux analyzer (Agilent Technologies, USA). Briefly, cells were seeded in Seahorse XFe96 well plates. One day before the assay, the hydration solution was added to the lower layer of the plate, which was then incubated overnight at 37 °C in a non-CO₂ incubator. On the following day, the cells were washed with pre-warmed Seahorse XF basal medium and covered with assay medium. The plate was kept at 37 °C without CO₂ for 1 h. Oligomycin (1 μM), FCCP (1 μM), rotenone/antimycin A (Rot/AA, 1 μM) were loaded into the injection ports of the sensor cartridge. For exogenous fatty acid oxidation assessment, palmitate-BSA or BSA control was added to designated wells. After 30 min, the hydrated sensor cartridge was aligned with the cell culture plate and inserted into the analyzer for measurement. Finally, the protein content in each well was quantified utilizing the BCA assay to normalize OCR.

**1.19 Separation of cytoplasmic and nuclear fraction**

Nuclear-cytoplasmic protein separation was performed using a commercial kit (Beyotime, China). After scraping the cells with PBS, they were centrifuged at 800 g and 4 °C for 5 min to collect the pellet. According to the kit protocol, cytoplasmic protein reagent was added to the pellet, followed by centrifugation at 16000g and 4 °C for 5 min, and the supernatant contained the cytoplasmic fraction. Nuclear protein reagent was then added to the remaining pellet, and after centrifugation at 16000g and 4 °C for 10 min, the supernatant was collected as the nuclear protein fraction.

**1.20 Protein-protein interaction analysis**

AlphaFold, GRAMM-X servers, and PDBePISA were used to dock CPT1A and PLIN2 following retrieval of the coding of the docked protein from Uniprot.

**1.21 Statistical analysis**

All data analyses were performed by GraphPad Prism (version 10.0, USA) and SPSS (version 26.0, USA). Student’s t-test was used to compare differences in continuous variables between two groups, while one-way ANOVA was employed for comparisons among three groups. The results were presented as mean ± SEM. P value < 0.05 was considered statistically significant.

**References**

[1] Yan X, Shen Z, Yu D, et al.Nrf2 contributes to the benefits of exercise interventions on age-related skeletal muscle disorder via regulating Drp1 stability and mitochondrial fission. Free Radic Biol Med 2021;17859-75.

[2] Wu M, Tan J, Cao Z, et al.Sirt5 improves cardiomyocytes fatty acid metabolism and ameliorates cardiac lipotoxicity in diabetic cardiomyopathy via CPT2 de-succinylation. Redox Biol 2024;73103184.


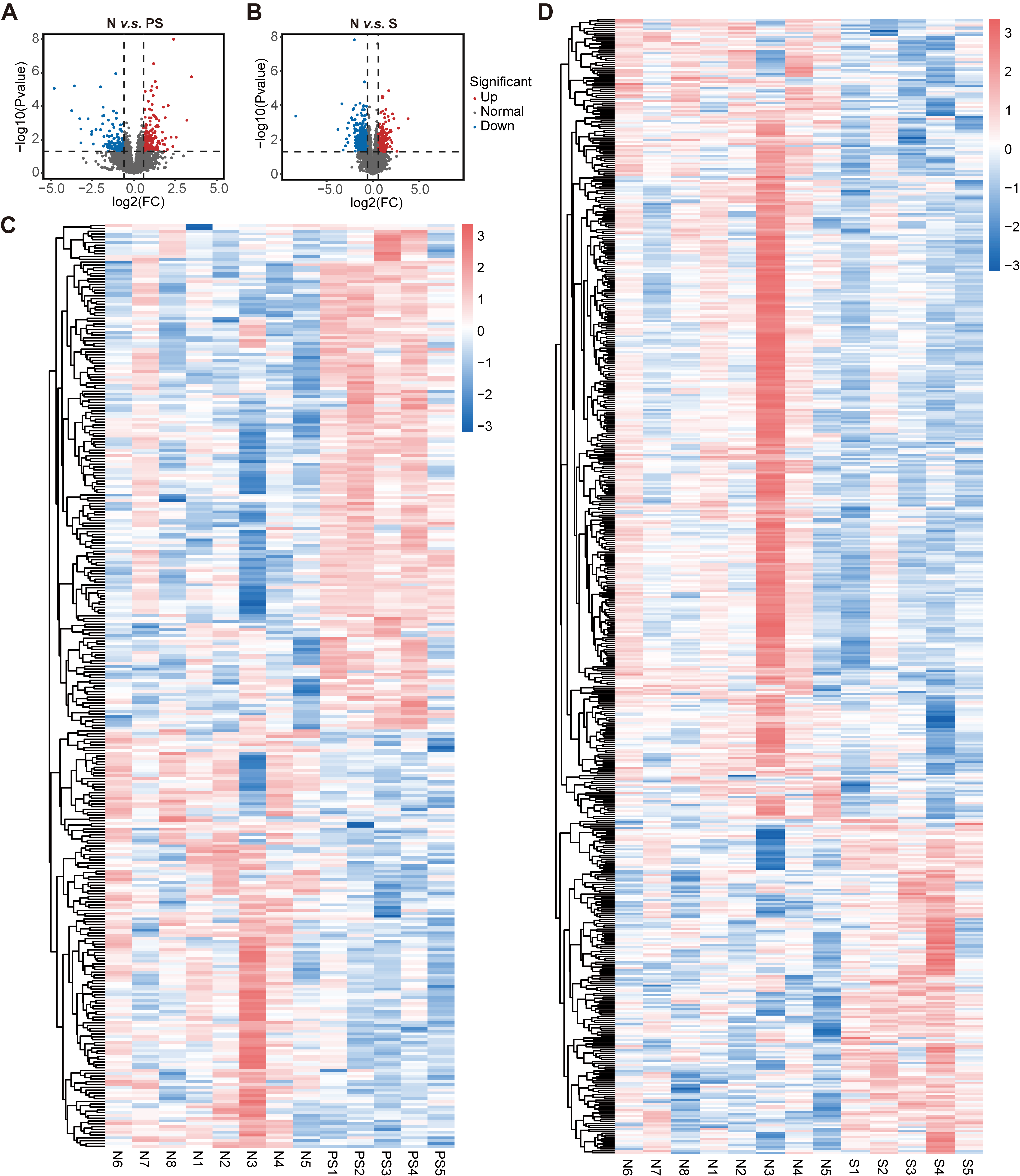


**Figure S1.** RNA sequencing of patients with non-sarcopenia (N), possible sarcopenia (PS), and sarcopenia (S). (A) Volcano plot of differentially expressed genes (DEGs)in skeletal muscle from non-sarcopenic and possible sarcopenic patients. (B) Volcano plot of DEGs in skeletal muscle from non-sarcopenic and sarcopenic patients. (C) Heatmap of DEGs clustering expression from non-sarcopenic and possible sarcopenic patients. (D) Heatmap of DEGs clustering expression from non-sarcopenic and sarcopenic patients.


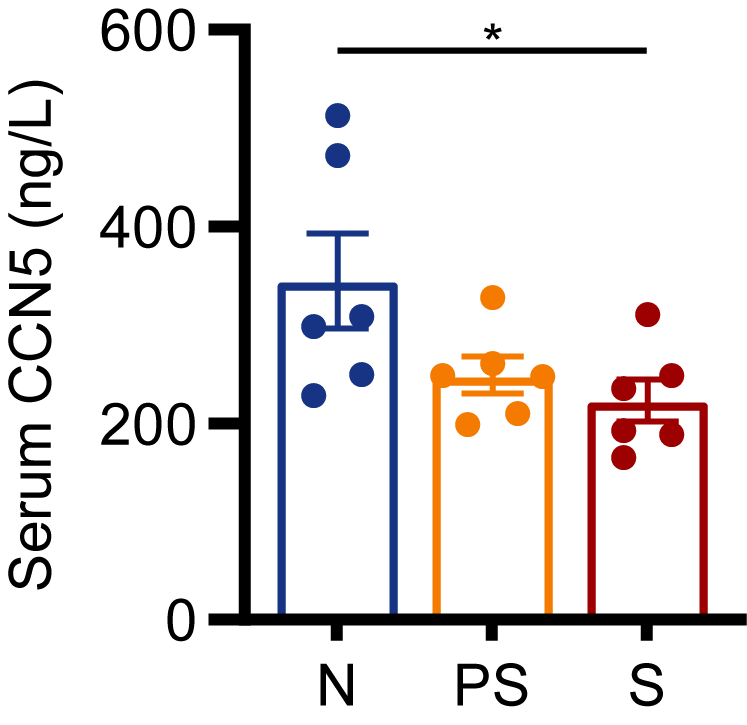


**Figure S2.** Serum levels of CCN5 from patients in N, PS, S groups (n = 6/group). The data were presented as mean ± SEM; ^*^*P* <0.05. N, non-sarcopenia; PS, possible sarcopenia; S, sarcopenia.


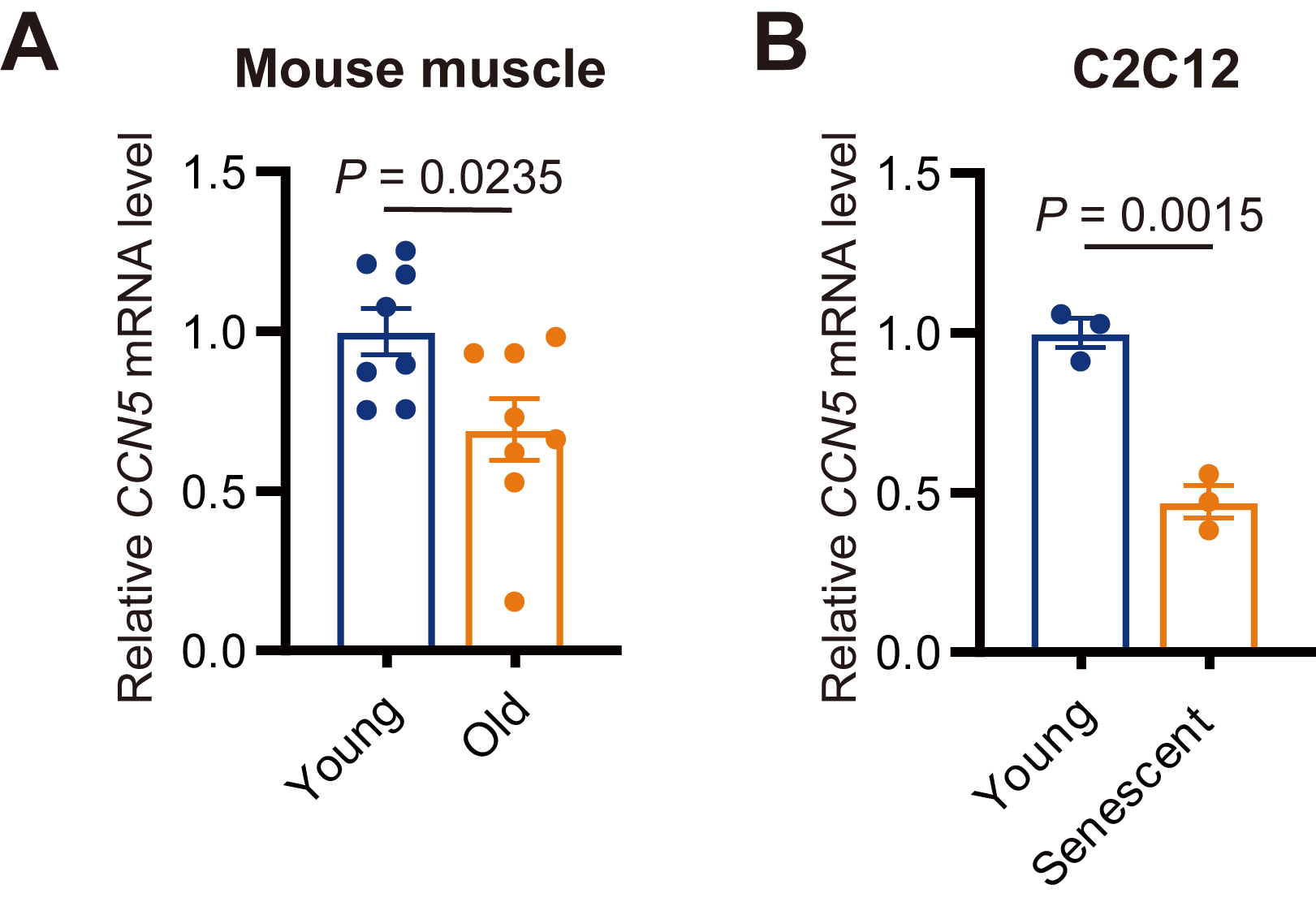


**Figure S3.** Validation of CCN5 mRNA expression levels in aged mice and aged myotubes. (A) qPCR analysis of CCN5 gene expression in muscles from indicated mice (n = 8/group). (B) qPCR analysis of CCN5 gene expression in young and senescent C2C12 cells (n = 3/group). The data were presented as mean ± SEM.


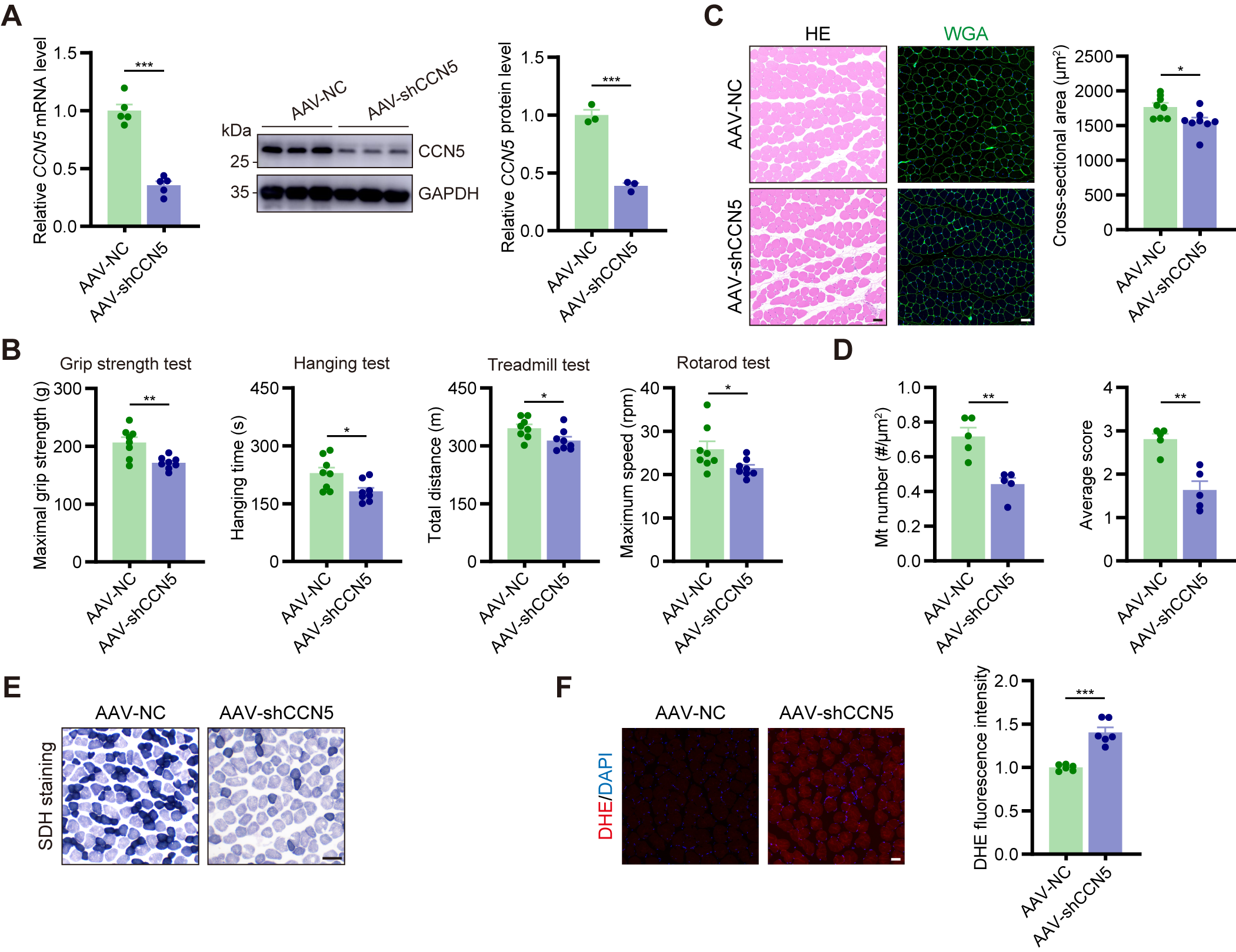


**Figure S4.** AAV9-shCCN5-mediated CCN5 downregulation impaired skeletal muscle function in young mice and decreased mitochondrial content and function. (A) Relative mRNA and protein expression levels of CCN5 in tibialis anterior (TA) muscle. (B) Maximal grip strength, four-paw hanging time, total distance for the treadmill test, maximum speed for the rotarod test of the mice in various groups. (C) HE and WGA staining of gastrocnemius (GA) and quantification of cross-sectional area (Scale bars: 50 μm). (D) The number and overall average score of mitochondria (Mt) and from TEM images. (E) SDH staining of GA sections (Scale bars: 100 μm). (F) Representative images of dihydroethidium (DHE) staining of GA muscle and quantitative data of reactive oxygen species (ROS) in various groups (Scale bars: 50 μm). The data were presented as mean ± SEM (n = 5-8/group); ^*^*P* <0.05, ^**^*P* <0.01, ^***^*P* <0.001.


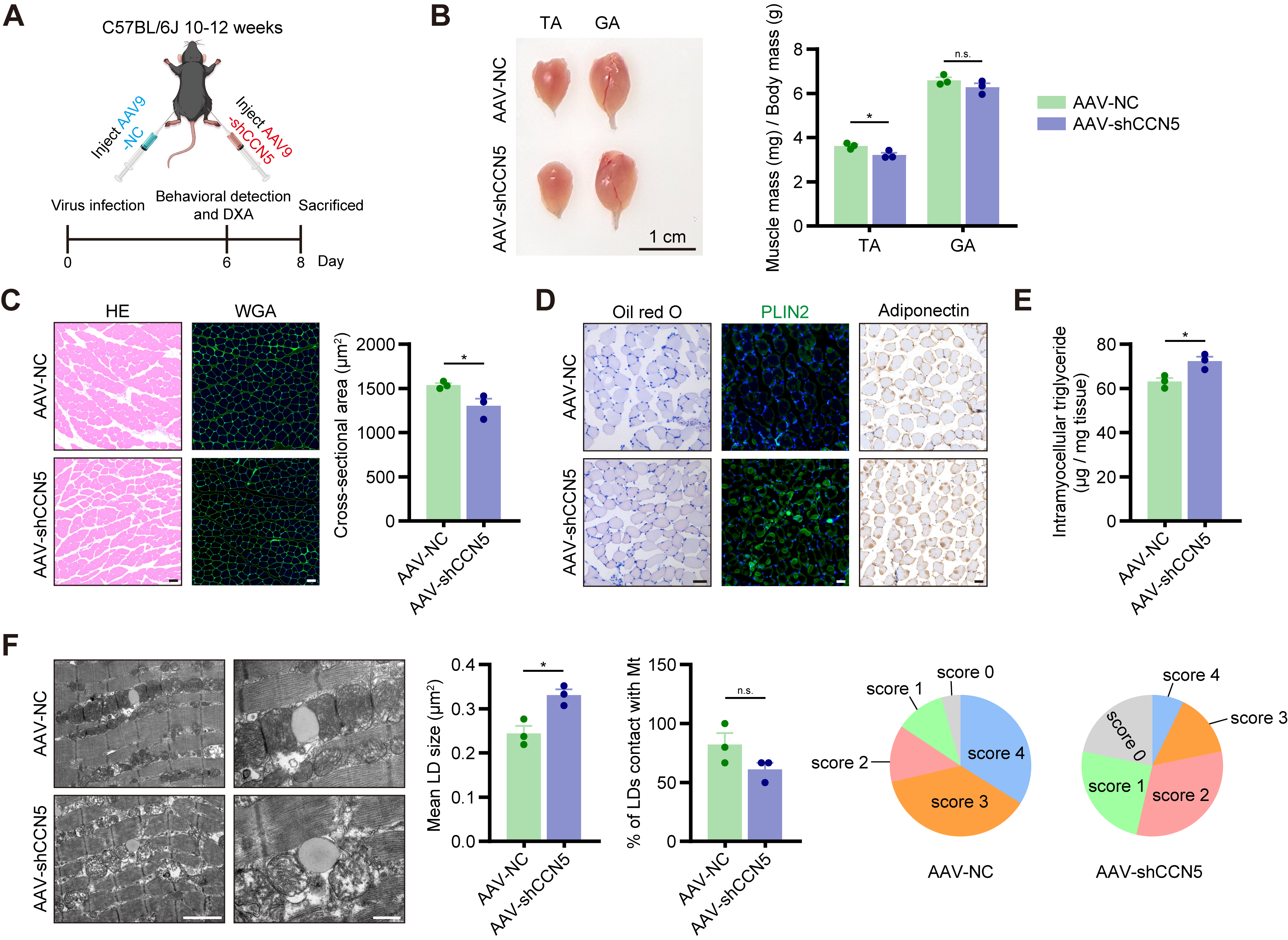


**Figure S5.** CCN5 reduction produced sarcopenia-like phenotype in young mice, especially myosteatosis. (A) Scheme of the experimental strategy. (B) Representative images and muscle mass of the tibialis anterior (TA) and gastrocnemius (GA). (C) HE and WGA staining of GA muscle and quantification of cross-sectional area (Scale bars: 50 μm). (D) Oil red O staining, immunofluorescent staining of PLIN2 and immunohistochemical staining of adiponectin of GA muscle (Scale bars: 50 μm). (E) Total triglyceride concentration in TA muscle. (F) Representative TEM images of lipid droplet (LD) and mitochondria (Mt) in GA muscle and quantification of LD, Mt (Scale bars: 2 (left) and 0.5 (right) μm). The data were presented as mean ± SEM (n = 3/group); n.s., not significant; ^*^*P* <0.05, ^**^*P* <0.01, ^***^*P* <0.001.


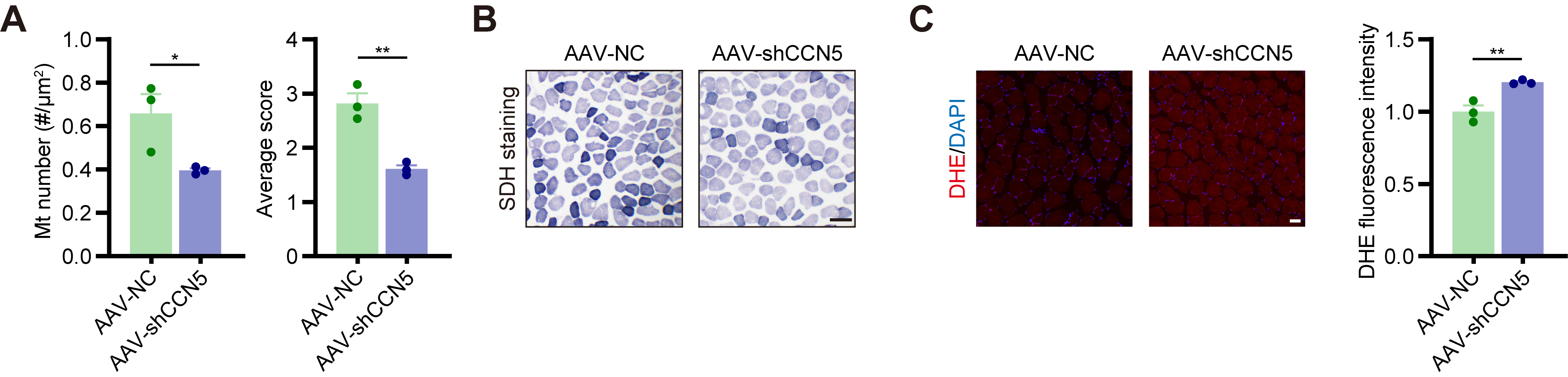


**Figure S6.** CCN5 reduction contributed to impaired mitochondrial content and function in young mice. (A) The number and overall average score of mitochondria (Mt) and from TEM images. (B) SDH staining of GA sections (Scale bars: 100 μm). (C) Representative images of dihydroethidium (DHE) staining of GA muscle and quantitative data of reactive oxygen species (ROS) in various groups (Scale bars: 50 μm). The data were presented as mean ± SEM (n = 3/group); ^*^*P* <0.05, ^**^*P* <0.01, ^***^*P* <0.001.


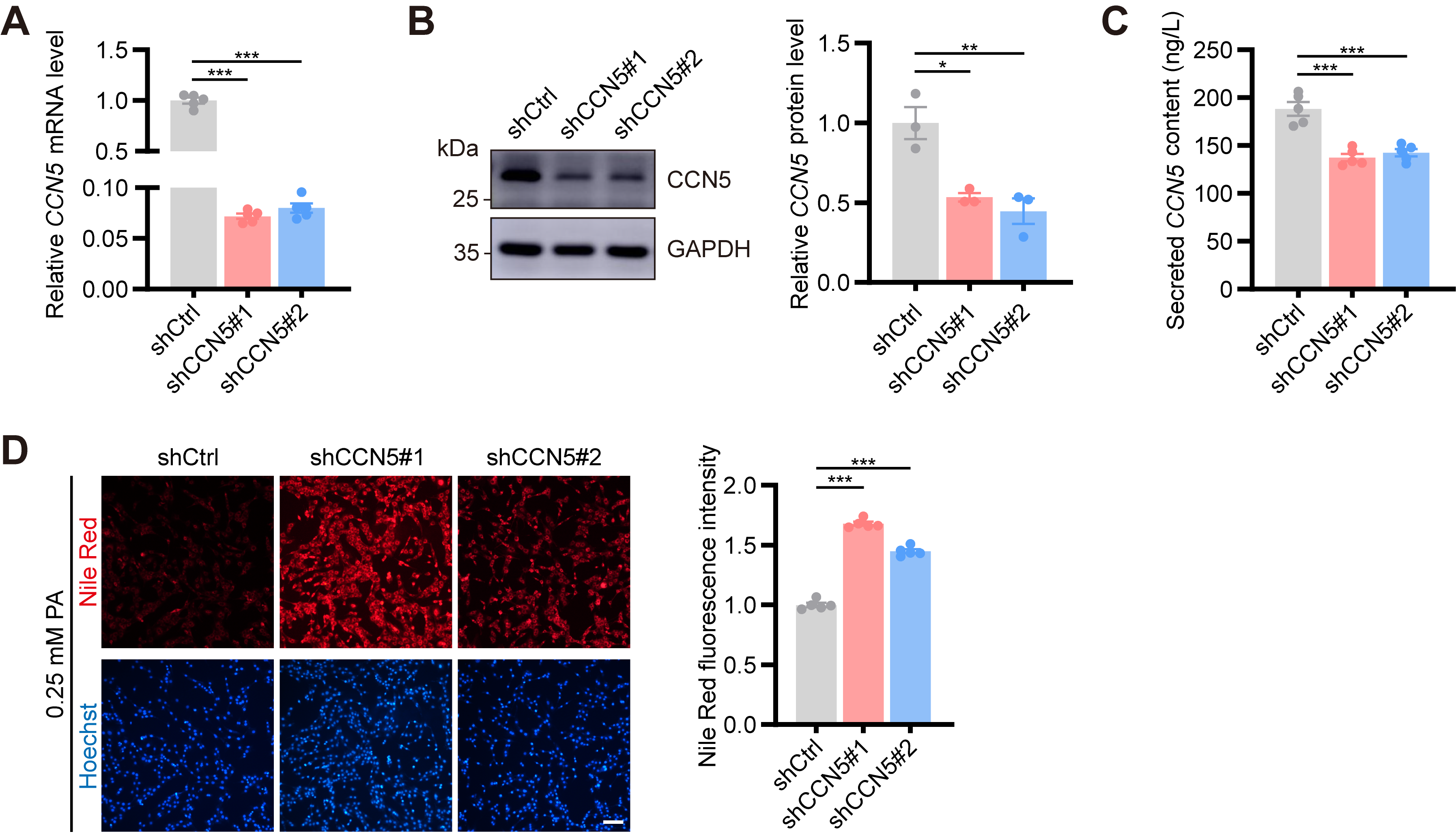


**Figure S7.** CCN5 deficiency induced lipid accumulation in C2C12 cells. (A) qPCR analysis of CCN5 gene expression in the indicated C2C12 cells (n = 5/group). (B) Relative protein expression levels and quantitative data of CCN5 in C2C12 cells with protein secretion blocked by 5 μg/ml Brefeldin A for 4 h (n = 3/group). (C) Secreted CCN5 expression level was determined by enzyme-linked immunosorbent assay (n = 5/group). (D) Nile Red staining and quantification of fluorescence intensity in C2C12 cells under 0.25 mM palmitate (PA) treatment (n = 5/group; Scale bars: 200 μm). The data were presented as mean ± SEM; ^*^*P* <0.05, ^**^*P* <0.01, ^***^*P* <0.001.


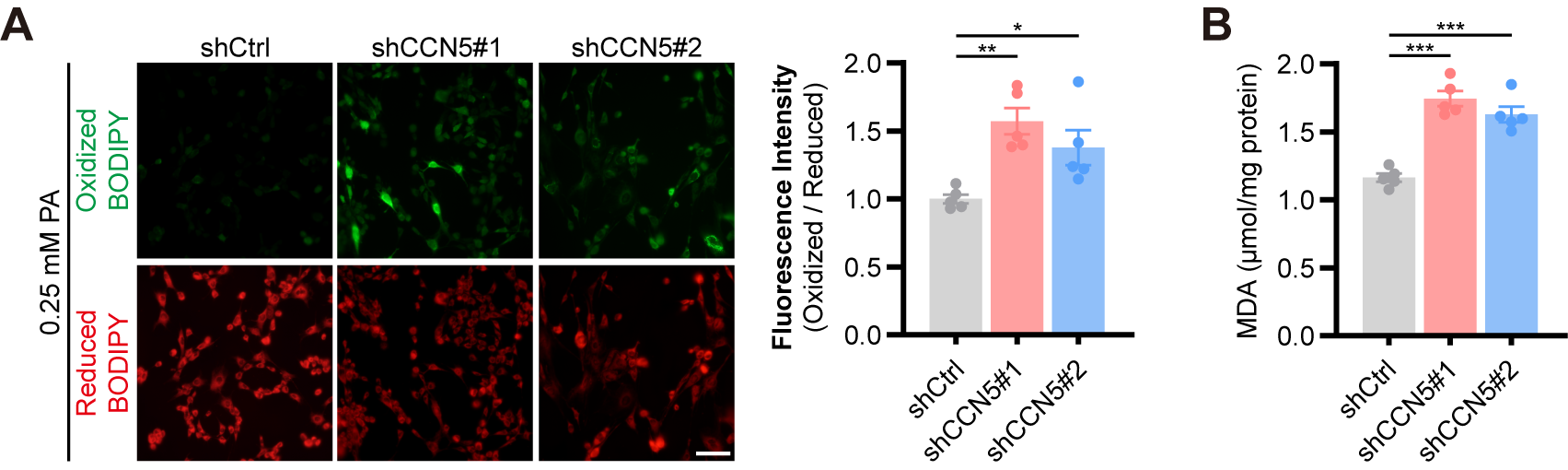


**Figure S8.** Increased lipid peroxidation in CCN5-deficient myotubes under PA treatment. (A) BODIPY staining and quantification of fluorescence intensity in C2C12 cells under 0.25 mM palmitate (PA) treatment (Scale bars: 100 μm). (B) Quantification of lipid peroxidation by MDA assay. The data were presented as mean ± SEM (n = 5/group); ^*^*P* <0.05, ^**^*P* <0.01, ^***^*P* <0.001.


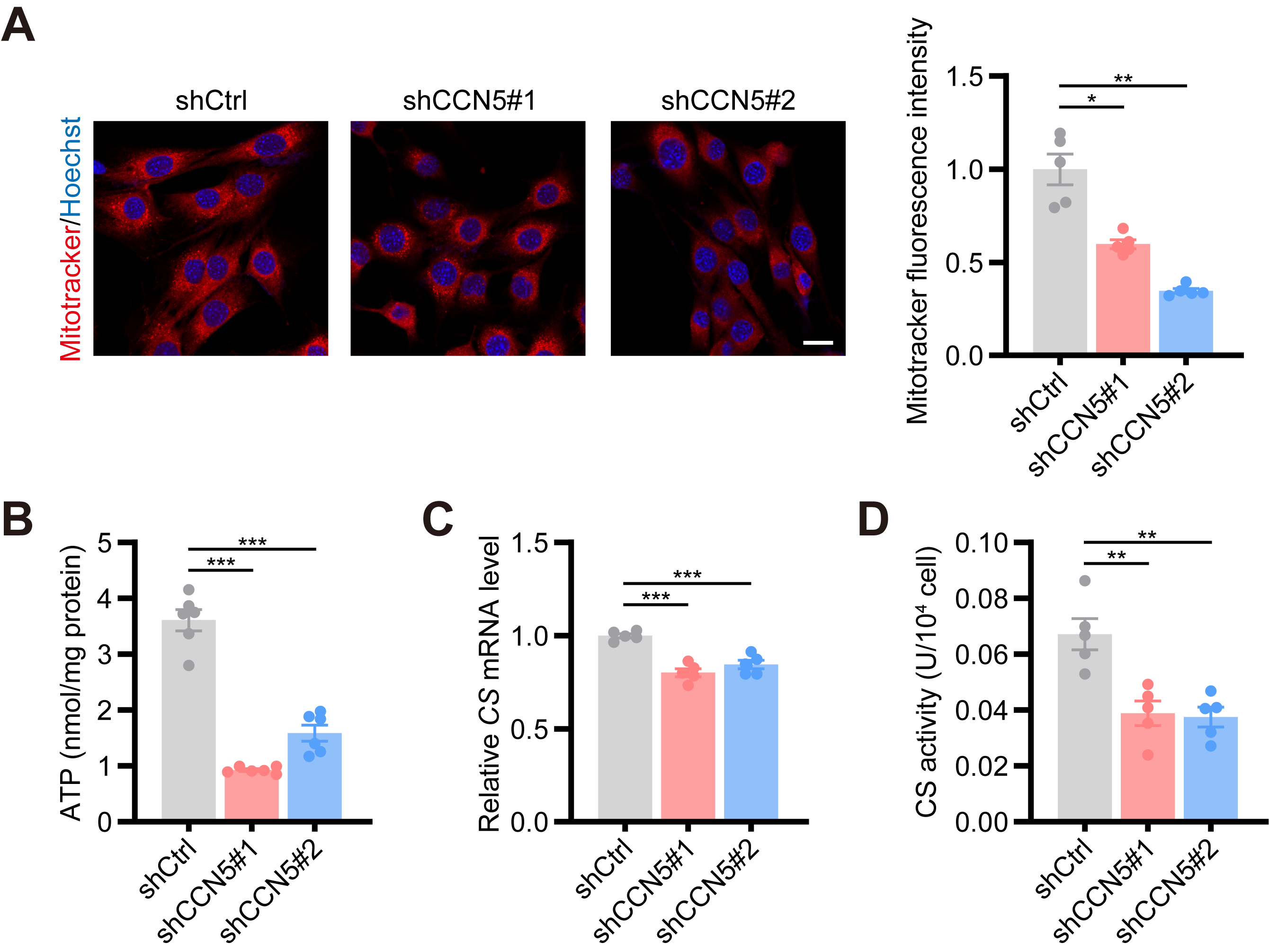


**Figure S9.** CCN5 expression impacted mitochondrial content and function in C2C12 cells. (A) Representative images of Mitotracker staining and quantification of fluorescence intensity (n = 5/group; Scale bars: 20 μm). (B) ATP content normalized by total protein content (n = 6/group). (C) Relative mRNA expression levels of citrate synthase (CS) (n = 5/group). (D) CS activity (by colorimetric method) of different groups (n = 5/group). The data were presented as mean ± SEM; ^*^*P* <0.05, ^**^*P* <0.01, ^***^*P* <0.001.


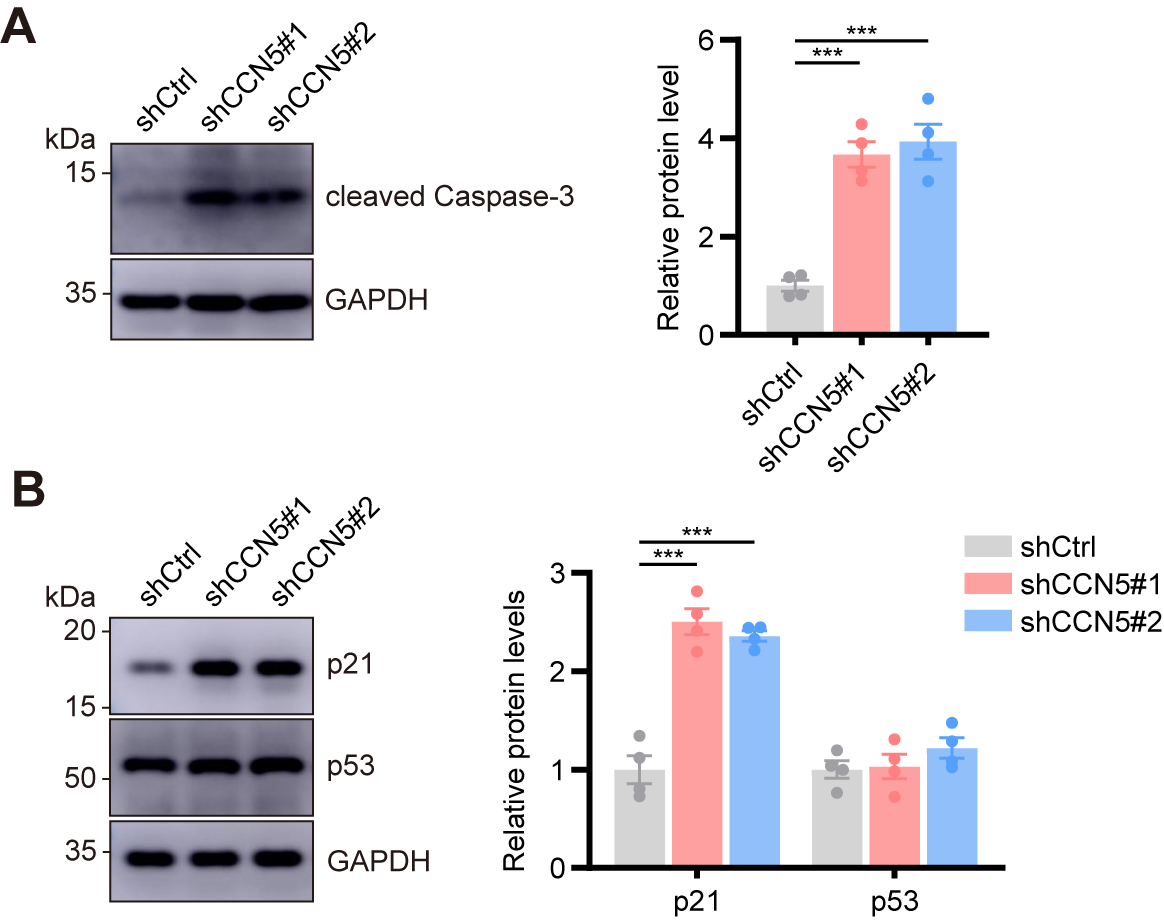


**Figure S10.** Analysis of apoptosis and senescence markers following CCN5 knockdown. (A) Relative protein expression levels and quantitative data of cleaved Caspase-3. (B) Relative protein expression levels and quantitative data of p21 and p53. The data were presented as mean ± SEM (n = 4/group); ^*^*P* <0.05, ^**^*P* <0.01, ^***^*P* <0.001.


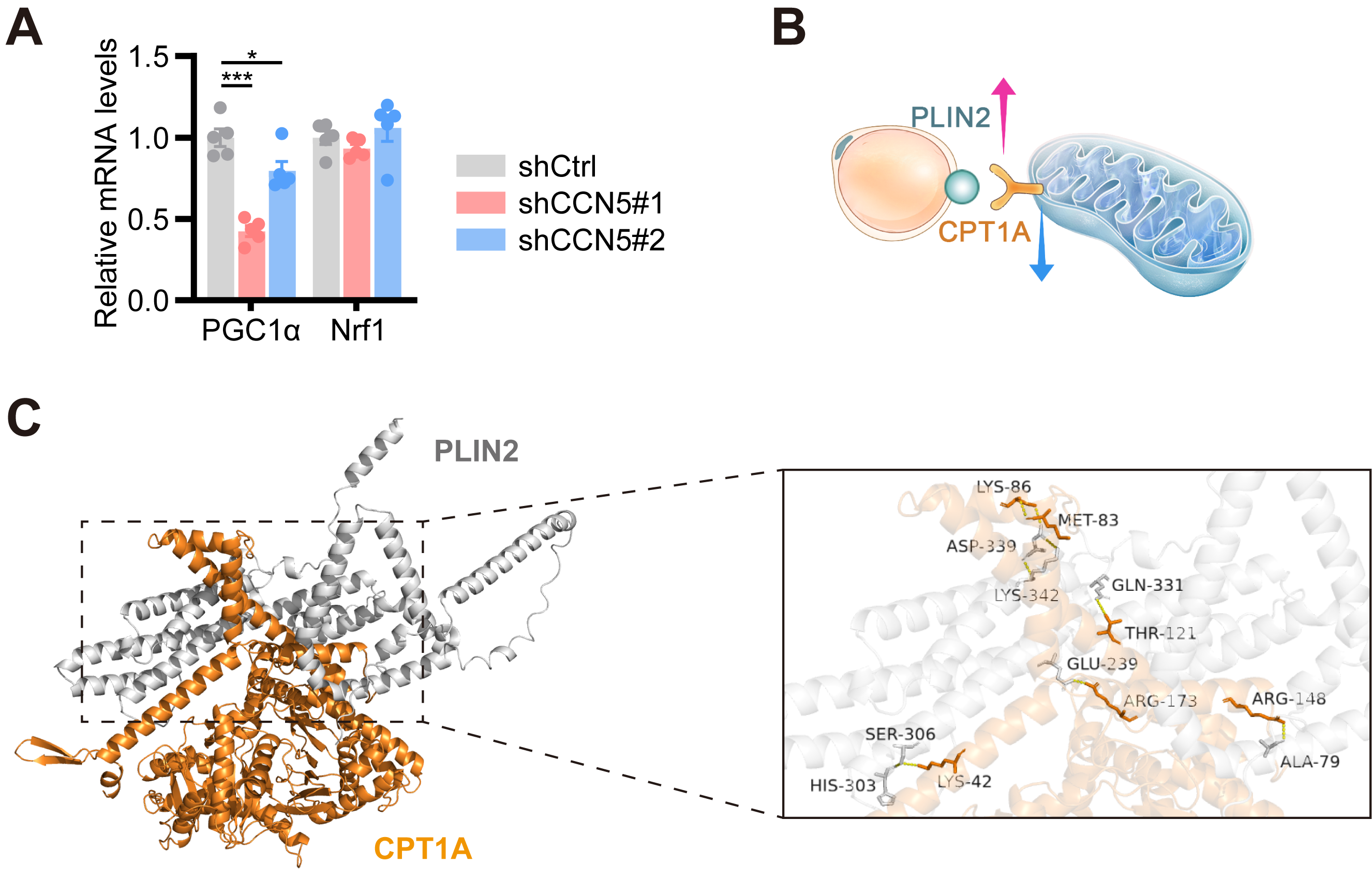


**Figure S11.** Secreted CCN5 enhanced FOXO3A-dependent transcription. (A) Relative mRNA expression levels of genes depended on FOXO3A transcription (n = 5/group). (B) Model of CPT1A-PLIN2 conjugation promoting lipid droplet-mitochondrial interactions. (C) Model diagram of the interaction between CPT1A and PLIN2 predicted by AlphaFold. The data were presented as mean ± SEM; ^*^*P* <0.05, ^**^*P* <0.01, ^***^*P* <0.001.


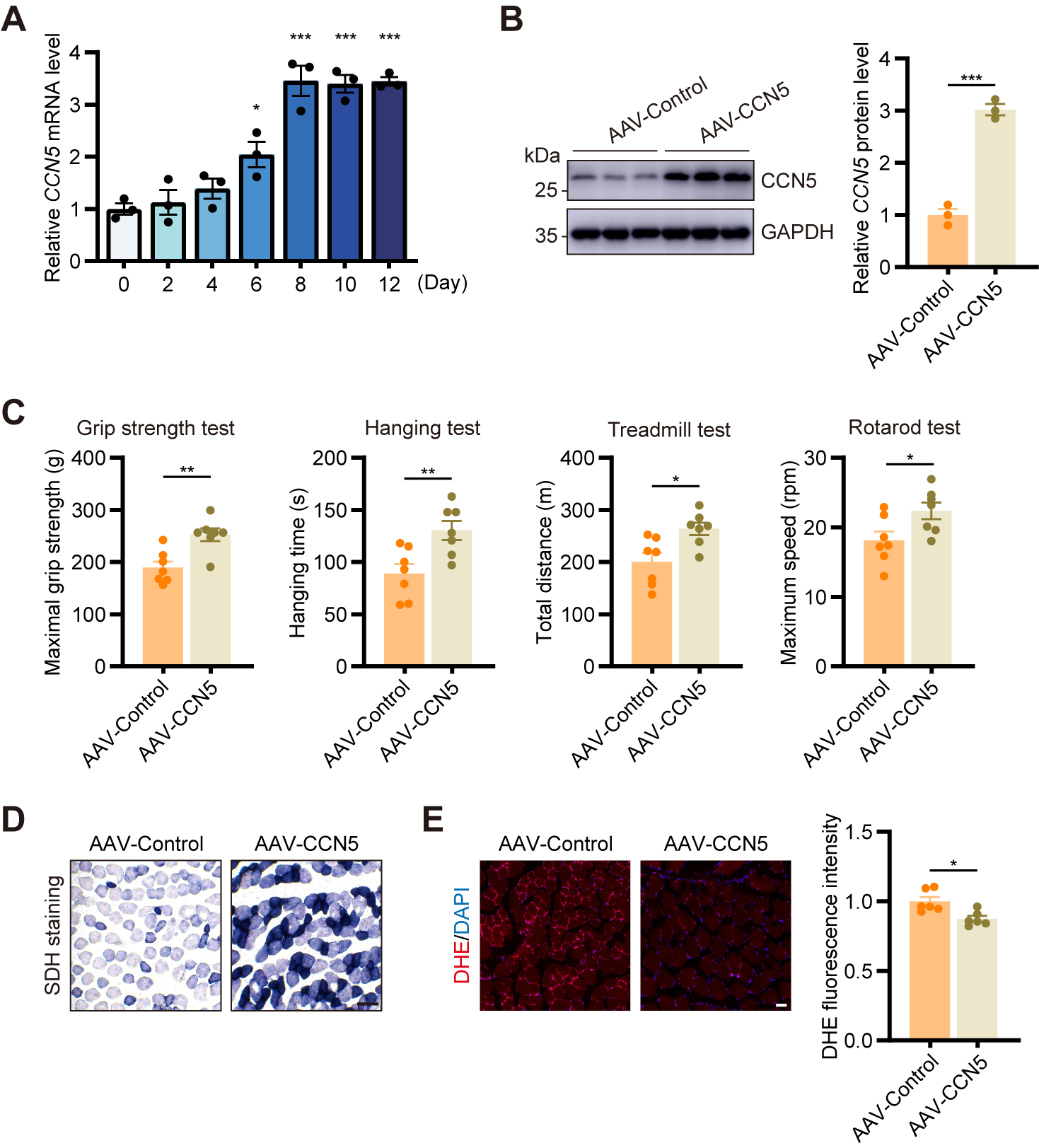


**Figure S12.** AAV9-CCN5-mediated CCN5 upregulation improved skeletal muscle function, enhanced mitochondrial content and function in aged mice. (A) Time-course analysis of CCN5 mRNA expression following intramuscular AAV injection (n = 3/group; statistical comparison vs. Day 0). (B) Relative protein expression levels and quantitative data of CCN5 in tibialis anterior (TA) muscle. (C) Maximal grip strength, four-paw hanging time, total distance for the treadmill test, maximum speed for the rotarod test of the mice in various groups. (D) SDH staining of GA sections (Scale bars: 100 μm). (E) Representative images of dihydroethidium (DHE) staining of GA muscle and quantitative data of reactive oxygen species (ROS) in various groups (Scale bars: 50 μm). The data were presented as mean ± SEM (n = 6-7/group); ^*^*P* <0.05, ^**^*P* <0.01, ^***^*P* <0.001.

**Table S1.** Clinical data of study patients by group.

| **Characteristic** | **Non-sarcopenia (n = 24)** | **Possible sarcopenia (n = 18)** | **Sarcopenia (n = 14)** | **P value** |
| --- | --- | --- | --- | --- |
| Age, mean ± SD, year | 58.54 ± 8.78 | 64.83 ± 5.95 | 69.14 ± 7.77 | < 0.001 |
| Men, n (%) | 12 (50.0%) | 7 (38.9%) | 10 (71.4%) | 0.183 |
| Height, mean ± SD, m | 1.65 ± 0.07 | 1.63 ± 0.09 | 1.65 ± 0.10 | 0.668 |
| Preoperative weight, mean ± SD, kg | 65.77 ± 10.25 | 69.41 ± 9.56 | 61.01 ± 12.65 | 0.098 |
| Preoperative BMI, mean ± SD, kg/m^2^ | 24.16 ± 3.04 | 26.16 ± 2.64 | 22.28 ± 4.33 | 0.007 |
| Calf circumference, mean ± SD, cm | 33.73 ± 2.65 | 34.96 ± 2.45 | 31.50 ± 3.08 | 0.003 |
| T12 SMI, median (IQR), cm^2^/m^2^ | 28.60 (27.34-35.94) | 31.61 (27.45-35.29) | 24.98 (20.61-27.89) | < 0.001 |
| Handgrip strength, median (IQR), kg | 30.45 (21.93-36.28) | 23.60 (15.93-26.80) | 19.40 (16.48-23.33) | 0.003 |
| Gait speed, mean ± SD, m/s | 1.21 ± 0.12 | 0.99 ± 0.16 | 0.93 ± 0.18 | < 0.001 |
| 5-time chair stand test, median (IQR), s | 9.00 (7.82-10.28) | 11.29 (9.05-12.79) | 10.32 (9.41-12.95) | 0.010 |
| SPPB, median (IQR), score | 12 (12-12) | 11 (10.75-12) | 11.5 (9.75-12) | 0.018 |
| TNM stage, n (%) |  |  |  | 0.181 |
| 0 | 9 (37.5%) | 2 (11.1%) | 4 (28.6%) |  |
| I | 12 (50.0%) | 14 (77.8%) | 5 (35.7%) |  |
| II | 1 (4.2%) | 1 (5.6%) | 2 (14.3%) |  |
| III | 2 (8.3%) | 1 (5.6%) | 3 (21.4%) |  |
| Laboratory data |  |  |  |  |
| C-reactive protein, median (IQR), mg/L | 3.15 (1.07, 3.33) | 3.34 (3.30, 3.84) | 5.66 (3.00, 14.91) | 0.012 |
| White blood cells, median (IQR), ×10^9^/L | 5.41 (4.49, 6.66) | 5.55 (4.88, 7.17) | 6.34 (5.39, 7.37) | 0.205 |
| Red blood cells, mean ± SD, ×10^12^/L | 4.64 ± 0.44 | 4.53 ± 0.57 | 4.31 ± 0.54 | 0.172 |
| Hemoglobin, mean ± SD, g/L | 136.67 ± 13.38 | 134.22 ± 11.99 | 130.07 ± 18.64 | 0.405 |
| Albumin, mean ± SD, g/L | 41.15 ± 3.89 | 39.68 ± 3.35 | 38.02 ± 3.74 | 0.046 |
| CAR, median (IQR) | 0.074 (0.026, 0.088) | 0.085 (0.078, 0.107) | 0.148 (0.078, 0.451) | 0.006 |

SD, standard deviation; BMI, body mass index; T12 SMI, skeletal muscle index at 12th thoracic vertebra; IQR, interquartile range; SPPB, Short Physical Performance Battery; TNM, tumor-nodule-metastasis; CAR, C-reactive protein-to-albumin ratio.

**Table S2.** The primary antibodies used for WB and IHC/IF assays.

| **Antibody** | **Supplier** | **Catalogue number** | **WB Dilution** | **IHC/IF Dilution** |
| --- | --- | --- | --- | --- |
| CCN5 | Abclonal | A7456 | 1:500 | 1:50 |
| GAPDH | Cell Signaling Technology | 2118 | 1:2000 | / |
| Laminin | Abcam | ab11575 | / | 1:50 |
| PLIN2 | Servicebio | GB115593 | / | 1:100 |
| Adiponectin | Servicebio | GB115581 | / | 1:50 |
| SREBP1 | Abclonal | A15586 | 1:500 | / |
| DGAT2 | HUABIO | ER60651 | 1:500 | / |
| CPT1A | Proteintech | 15184-1-AP | 1:1000 | / |
| ACOX1 | Abclonal | A21217 | 1:2000 | / |
| SCAD | HUABIO | HA721058 | 1:500 | / |
| MCAD | HUABIO | ET7109-74 | 1:500 | / |
| LCAD | HUABIO | ER1901-11 | 1:500 | / |
| HSL | Abclonal | A15686 | 1:500 | / |
| PLIN2 | Abclonal | A24464 | 1:3000 | / |
| Tom20 | Cell Signaling Technology | 42406 | 1:1000 | / |
| Tubulin | Cell Signaling Technology | 2125 | 1:2000 | / |
| NDUFB8 | Affinity | DF9666 | 1:1000 | / |
| SDHB | Affinity | DF12732 | 1:500 | / |
| UQCRC2 | Affinity | DF12339 | 1:500 | / |
| MTCO1 | Abcam | ab14705 | 1:1000 | / |
| ATP5A | Affinity | DF3806 | 1:500 | / |
| p21 | Abclonal | A22460 | 1:1000 | / |
| p53 | Abmart | TA0879F | 1:1000 | / |
| β-Catenin | Proteintech | 51067-2-AP | 1:5000 | 1:50 |
| Lamin B1 | HUABIO | ET1606-27 | 1:50000 | / |
| FOXO3A | Proteintech | 66428-1-Ig | 1:2000 | 1:50 |
| ZFP423 | Abclonal | A15795 | 1:500 | / |
| PPARγ | Affinity | AF6284 | 1:500 | / |

**Table S3.** Primer sequences for RT-qPCR analysis.

| **Gene** | **Forward primer** | **Reverse primer** |
| --- | --- | --- |
| *Human*-GAPDH | GGAGCGAGATCCCTCCAAAAT | GGCTGTTGTCATACTTCTCATGG |
| *Human*-PVALB | ACTTGCTGAACGCTGAGGACATC | ACCTTCTTCACATCATCCGCACTC |
| *Human*-MYL5 | CGCCGAGGAGACCATTCTTAACG | CTCTTCCGCCGTCATCTTGTCAG |
| *Human*-IL32 | TGTGCTTCCCGAAGGTCCTCTC | TCTGCCAGGCTCGACATCACC |
| *Human*-FASN | GTGGTGGGCTTGGTGAACTGTC | AGGTGCTGCTGAGGTTGGAGAG |
| *Human*-SHISA2 | CCTCATTGTTGGCTCCGTGTTTG | CTGTGCTGGACTGGCGTGAG |
| *Human*-CCN5 | CACGCATAGGCTTGTATTCAGGAAC | CACGCTGCCTGGTCTGTCTGGATC |
| *Human*-MYLK4 | ATCTGGACATAATGAGGCGAAGGAG | TTGCTTGGCTGTCACAATACGATG |
| *Human*-MIDN | CGCAGCGACAGCAGTAGCAG | GCCACCACGAACTCTGACTTGATG |
| *Human*-SLC41A1 | GATTCTCCTGTACATCGCAGAC | CCCCTATGAGCCAGAGAACA |
| *Human*-PFKFB2 | CGGCGTGAAGCAGTCAAGTCC | CACCGCAATCTGACCATTCTCCTC |
| *Mouse*-GAPDH | TTCAACAGCAACTCCCACTCTT | TGGTCCAGGGTTTCTTACTCC |
| *Mouse*-CCN5 | CGCTGTGATGACGGTGGTTTC | ACCTTCCTGGCACCTGTATTCTC |
| *Mouse*-FATP1 | CCGTATCCTCACGCATGTGT | CTCCATCGTGTCCTCATTGAC |
| *Mouse*-FATP2 | GATGCCGTGTCCGTCTTTTAC | GACTTCAGACCTCCACGACTC |
| *Mouse*-SREBP1 | CTTACCCCTCCACCCTCAGA | TGTCGGGCTCAGAGTCACTA |
| *Mouse*-DGAT2 | GAAGATGTCTTGGAGGGCTG | CGCAGCGAAAACAAGAATAA |
| *Mouse*-CPT1A | CATCGTGAGTGGCGTCCTCTTTG | AGTGCTGTCATGCGTTGGAAGTC |
| *Mouse*-ACOX1 | CCGTCGAGAAATCGAGAACT | ATTGAGGCCAACAGGTTCCA |
| *Mouse*-SCAD | ATGTGCCAGAGGAGCTGAGT | TGATCCACTGTTGCTTCTGC |
| *Mouse*-MCAD | AACTAAACATGGGCCAGCGA | CAGCTGCGACTGTAGGTCTG |
| *Mouse*-LCAD | GCATCAACATCGCAGAGAAA | ACGCTTGCTCTTCCCAAGTA |
| *Mouse*-HSL | CCGCTGACTTCCTGCAAGAG | CTGGGTCTATGGCGAATCGG |
| *Mouse*-COX4 | ACTACCCCTTGCCTGATGTG | GCCCACAACTGTCTTCCATT |
| *Mouse*-mtDNA | CCGCAAGGGAAAGATGAAAGA | TCGTTTGGTTTCGGGGTTTC |
| *Mouse*-nDNA | CCCTGTCATGTCCCTTTGTT | GCCACCAGCTCAGTTAAAGG |
| *Mouse*-CS | CAAGTCATCTACGCCAGGGACA | CAAAGCGTCTCCAGCTAACCAAG |
| *Mouse*-PGC1α | GTGCCACCGCCAACCAAGAG | TTCCTCGTGTCCTCGGCTGAG |
| *Mouse*-Nrf1 | AAACAAAGGGTTTCATGGAC | GGTACGAGATGAGCTATACTG |
| *Mouse*-PPARγ | GTACTGTCGGTTTCAGAAGTGCC | ATCTCCGCCAACAGCTTCTCCT |
